# Treatise on the Formation, Constituents, and Extraction of the Urinary Calculus, &c. &c.

**Published:** 1835-04-01

**Authors:** 


Medico-Chirurgical Review,

No. XLIV.

[No. 4 of a Decennial Series.]

JANUARY 1, to APRIL 1, 1835.

### A Treatise on the Formation, Constituents, and Extraction of the Urinary Calculus; being the Essay for which the Jacksonian Prize, for the Year 1833, was awarded by the Royal College of Surgeons in London. By *John Green Crosse,* Surgeon to the Norfolk and Norwich Hospital, and Lecturer on Clinical Surgery, &c. &c. &c. 4to, pp. 231, with 29 coloured Lithographic Plates. Churchill, London, 1835.

The present work essentially consists of a systematic treatise on the causes, composition, and consequences of renal and vesical calculi, and on the operations required for their removal; and of three appendices severally devoted to the following subjects—cases of litho-cystotomy—tables shewing the results of operations of litho-cystotomy, chiefly performed in the Norfolk and Norwich Hospital—a catalogue of the express treatises upon gravel, stone, and lithotomy, published in different ages and countries, and of essays or notices referring to those subjects in many periodical and some other works.

Mr. Crosse informs us, in two advertisements or prefaces, that the text of the present Treatise is the Prize-Essay presented to the Royal College of Surgeons of London, with only a very few verbal alterations and corrections. The notes to each chapter were added after the work was in the printer's hands, and the three appendices are of course additions. The Prize-Essay itself, the foundation of the whole, was commenced and completed in the brief time intervening between the latter end of October and the middle of December 1833, and even that brief time was rendered less available by the constant interruptions arising from an extensive private practice.

Such are the apologies for the execution of the work, which the author offers, and he diffidently observes that he can scarcely hope that what he has penned will bear strict inspection, either as to its composition or arrangement. The industry and the ability of Mr. Crosse may allow the reader to dissent from the estimate which his modesty has formed, and to anticipate with pleasure the perusal of the results of his study and experience.

The diseases of the urinary organs have attracted of late so much attention, that the public, we mean the professional public, must despair of seeing much novelty at present. Yet every labourer does something in the field, and the aggregate of benefit is far from inconsiderable. The judicious surgeon derives some advantage from the observations and reflections of various authors, some of whom see more than others, but each of whom will probably have noticed something that escaped the rest. Mr. Crosse may be assured that he derives no discredit from toiling in the same vineyard with Brodie and Prout. We think we need not occupy the time of our readers with any further introductory remarks, and we therefore proceed to the consideration of the first chapter of the work. This consists of some remarks on the causes of urinary calculi.

Mr. Crosse observes that he does not attempt to enter fully into the causes which give rise to urinary calculi. The two most important may be fairly admitted to be dyspepsia, and disease in some part of the urinary apparatus. Both act upon the urine as a chemical fluid—dyspepsia affecting its components as a secretion, local changes disturbing it after it is secreted.

Dyspepsia may be occasioned in various ways, but its usual, or, at all events, its frequent tendency is to induce a superabundance of acid in the stomach, and of acid also (the lithic) in the urine. Dyspepsia is so much more frequent than are calculous disorders, that probably the assistance of other agencies is commonly required to produce them. Those ministering causes are want of exercise, variable climate, peculiar diathesis, articles of diet or of medicine, and, as has been stated, local disease. Before we advert more particularly to the latter, we may glance for a moment at the influence of climate and of food.

Calculous diseases have been *said* to be extremely rare in hot climates. Yet a glance at the last volume of the Transactions of the Medical Society of Calcutta, will show that in Hindostan many cases of stone in the bladder occur.* There appears to be less doubt of their infrequency in very cold countries, and the temperate zone is that in which alone they may be said to prevail. The sympathy which exists between the kidneys and the skin is potent and familiar. The most sudden, if not the most extreme variations of temperature, take place in the temperate zone—the repressed secretions of the surface demand increased exertions of the kidneys, and calls so sudden and so frequent may be reasonably supposed to render them additionally liable to disease.

It has been observed with natural surprise, that certain parts of certain countries are famous for the prevalence of calculous complaints. The diet, the soil, the waters have been accused, with some semblance of reason, but without satisfactory proof. The county of Norfolk is in this predicament. Mr. Crosse indulges in the following opinions in the way of explanation.

"In the county of Norfolk, where undoubtedly calculous disorders are very prevalent, perhaps even more so than in any other district of the United Kingdom, neither the food, soil, nor beverage, so far as I have been able to ascertain, have any particular share in determining such a result. Minute observations upon the climate might better explain the matter; the great prevalence of a north-east wind, and the frequent, sudden, and very considerable changes of temperature, acting upon persons already affected with the most prevailing

---

* A reference will be found to these in our last and the preceding number.—*Eds.*

disorders of the district, dyspepsia, scrofula, or rheumatism, cannot but be regarded as most powerful agents in giving rise to such frequent cases of gravel and stone. I have repeatedly known persons, who were free from gravelly complaints whilst residing in the metropolis, affected by them on spending a few weeks in the county referred to, and relieved, or entirely freed from them on a change of residence, although in each situation they followed carefully the same diet. Can we disregard climate, and the peculiar state of the atmosphere, in our attempts to explain such effects? 3.

It is probably a combination of circumstances which occasions an insalubrity of this description. The inquisitive observer might perhaps discover, in other portions of Great Britain, a keener air than is felt in Norfolk, unattended, notwithstanding, with a corresponding frequency of instances of stone.

The effect of diet is more obvious and immediate on the composition of the urine, than are those recondite agencies to which we have alluded. Luxurious living disposes to the generation of acid, and to gout. The indulgence in acids, or in spirituous liquors, speedily gives rise to crystals of lithic acid in the urine; and, pari passu, alkalies determine an alkaline condition of it. There is a fact connected with the operation of food in these cases, to which Mr. Crosse has not adverted. Sir Benjamin Brodie has remarked, that calculous diseases are most frequent in children of the lower classes, and in adults or elderly individuals of the upper ranks. The reason would appear to be this:—That in the lower class the children are ill-fed, ill-clothed, and exposed to all the causes which disorder the digestive organs; while adults, in the better classes, commit those excesses which produce the same results. There can be little question that this explanation approaches the truth. The fact is certainly important.

Local disease, more particularly stricture, and enlargement of the prostate gland, are powerful agents in the production of stone. Whatever interrupts the free evacuation of the urine must tend to this result; for, upon the one hand, the mere accumulation of the urine induces a disposition to the precipitation of its salts; and, on the other, inflammation of the mucous membrane of the bladder, ureter, and kidneys is excited, which, of itself, occasions depositions of the phosphates. A blow upon the loins, injuring the structure of the kidney, or deranging its functions, has been often known to give rise to calculous formations. Whatever creates inflammation of the lining membrane of the pelvis of the kidney, will usually induce the same effects, more especially when the diseased action leads to a preternatural mucous secretion from the surfaces over which the urine, after escaping from the tubuli, passes, in its course towards the bladder.

" Hernial displacement of the bladder, sacculi formed by its inner membrane passing between the fibres of the muscular coat, and prolapsus of the organ (which occurs only in the female), are so many disposing causes to stone, by creating dysuria, giving rise to an inflammatory and catarrhal state of the bladder, and detaining a quantity of urine constantly within some part of its cavity.

Extraneous substances, as a particle of lymph or of coagulated blood, a portion of bougie, or any other foreign body introduced from without, placed in any of the urinary cavities, so that the urine comes in contact with it, will become the nucleus of a calcareous deposit in any constitution, and in a torrid as well as temperate climate. Such concretions around an extraneous substance are usually alkaline, and there is reason to believe that they are not furnished exclusively by the urine, as it is discharged from the tubuli, but in some measure by the morbid secretion from the lining mucous membrane, whether of pelvis of the kidney, or of bladder, in consequence of the irritation and chronic inflammation excited by the extraneous substance.

Sedentary habits rank amongst the circumstances disposing to urinary concretions, by favouring indigestion, gout, and enlargement of the prostate gland: and where dyspepsia is present, all the causes enumerated act with greater force, and the result is produced with most certainty, by two or more causes combining together." 6.

### OF THE CHEMICAL COMPOSITION OF URINARY CALCULI.

Such is the title of the second chapter. We need not do more than allude to a point or two connected with the subject, the standard works on chemical science supplying the requisite information to all. But Mr. Crosse properly insists on the paramount necessity of investigating the structure of the nuclei of stones. Calculi which resemble them in size and composition often pass away spontaneously, and, under such circumstances, the condition of the urine acquires a paramount degree of importance, and an acquaintance with it is rendered absolutely necessary, to enable us to give effect to our preventive remedies. Actuated by this conviction, Mr. Crosse began, several years ago, to collect and examine all urinary concretions voided by the urethra. The following is the analysis of the first hundred, obtained from the male, and placed in the cabinet of Mr. Crosse.

| | |
|---|---|
| Lithic acid, or lithate of ammonia .... | 72 |
| Lithic acid and oxalate of lime........ | 9 |
| Oxalate of lime.................... | 14 |
| Carbonate of lime.................. | 1 |
| Triple phosphate .................. | 2 |
| Fusible ........................... | 2 |
| Total.................. | 100 |

Mr. C. observes that the frequency of small stones voided by the urethra, affords great opportunities of collection and examination. He remarks, also, on the large proportion of calculi, in the above table, containing lithic acid, and on the fact, that oxalate of lime should be met with in so many as twenty-three out of a hundred. His directions for the analysis of calculi of oxalate and carbonate of lime are deserving of consideration.

"The analysis of oxalate of lime is one of the simplest of chemical processes, and can be effectually done upon the smallest portion, even the hundredth part of a grain, so as readily to detect its presence. A particle of this concretion, being submitted to the flame of a spirit-lamp, urged by the common blow-pipe, a drop of dilute nitric acid is applied to the residue, and immediately globules of air are extricated, and can be seen rising through the fluid with a magnifying-glass, or even with the naked eye; these globules of air are carbonic acid gas, the heat applied having been just sufficient to decompose the oxalic acid, and out of its elements carbonic acid gas was formed, which united with the lime.

Carbonate of lime, a very rare form of human urinary concretion, gives out globules of carbonic acid gas, on the addition of dilute nitric acid, before the heat of a spirit-lamp has been applied, which distinguishes it from oxalate of lime, upon which dilute nitric acid produces no effervescence, until by heat the chemical changes just described have taken place.

The correction of one error of a writer of high authority is nearly equal to the statement of a new fact, which is my reason for offering these brief remarks upon a simple test for oxalate of lime, Dr. Marcet* having stated that when a portion of this concretion is submitted to a gentle heat, the oxalic acid is drawn off and pure lime left; but this change will not happen, unless an intense heat be applied, the flame of a spirit-lamp, urged by the common blow-pipe, producing only the changes I have described, and leaving carbonate of lime." 9.

The third chapter is devoted to the—

### Mechanical Composition and Growth of Vesical Urinary Calculi.

We need not dwell on the specific gravity of various calculi. Those composed of oxalate of lime are the heaviest, and usually the hardest—next to these are the stones of lithic acid—and phosphatic calculi are the lightest and the softest. But calculi of lithic acid or lithate of ammonia, with a little oxalate of lime, are sometimes remarkably friable, and occasionally break in the bladder from violence in sounding, or, when there are several, by knocking against each other. Mr. Crosse refers to some instances of this description. He conceives that some angular urinary calculi, with flat surfaces resembling biliary calculi, are the consequence of fracture. He removed thirty from the bladder of one patient; they were composed of lithic acid and oxalate of lime.

The ratio of increase of a calculus, when formed and retained in the bladder, is a subject of much importance to the surgeon and the patient. Such a calculus usually continues to increase in size, whilst the latter lives to be tortured by its presence. It is probable, however, that if the patient's health is good, his urine maintained in a natural state, and his bladder not affected by disease, the stone may remain for some time stationary in its size. The facts on this point require deliberate consideration. One only has come under the immediate observation of our author. It was the case of a patient who, twenty years before his death, was assured by his surgeon, after sounding, that he had a stone; he refused the operation, and at the end of that number of years died, and the stone found in his bladder weighed only six drachms and one scruple, and was composed of almost pure lithic acid.

If any calculi in the bladder can continue stationary, those of lithic acid or oxalate of lime may be expected, à priori, to do so; for their growth is slowest, and they are accompanied by the least amount of disease in the lining membrane of the bladder.

"In general, calculi when once formed go on increasing; and the estimate I venture to make, from a careful comparison, in a vast many instances, between the duration of the symptoms and the size of the stone removed by cystotomy, is that a concretion of lithic acid or oxalate of lime will, in an adult, usually grow between one and two drachms in a year, rarely exceeding the latter. The actual increase, in all probability, will be greater, the larger the stone and more extensive the surface presented; but I have never found reason to believe that, in calculi of moderate size, above four drachms have been deposited in a year; the largest vesical calculi I have met with, weighing from eight to twelve ounces,

* Marcet's Essay on Calculous Disorders, 1817, p. 121.

have been fifteen or twenty years in forming. The following case demonstrates the rate of increase with some accuracy in a particular instance.

A patient was lithotomized, and two lithic acid calculi, weighing seven drachms and a half, were removed, one of which broke into several portions under pressure of the forceps. The patient recovered from the operation, but soon had a recurrence of symptoms of stone, which he bore for above seven years, when he died aged seventy-six. The calculus found in his bladder weighed ℥ij. ʒij. ℈j., and presented on a section a clear exhibition of a portion of the calculus left in the bladder at the time he was lithotomized, and which formed the nucleus of the subsequent deposit. The calculus is composed of lithic acid, and about two ounces were deposited in the period of seven years and a half." 12.

The inexperienced surgeon must reflect, that calculations of the probable increase of calculous concretions, in any determinate period, are liable to be affected by great disturbing circumstances. The presence of a lithic stone in the bladder may give rise to inflammation and disease of that organ, or, deteriorating the health, may lead to the secretion of alkaline urine. In both cases, the phosphates are deposited, and the augmentation of the calculus is more rapid.

Phosphatic concretions, whether primary, or consecutive on lithic nuclei, are in general of quick growth, and never, perhaps, remain absolutely stationary. Mr. Crosse successfully lithotomized a gentleman, who had suffered symptoms of stone only between three and four months; the concretion had taken place upon a soft nucleus of mucus, under a very morbid and catarrhal state of the bladder, with the prostate gland a little enlarged, and a stricture of the urethra. The calculus was so brittle, that it broke into a hundred pieces; and the fragments weighed above four drachms and a half. The soft nucleus occupied so large a space, that the calculus must have measured an inch in diameter; its composition was fusible, with a slight trace of lithic acid.

It may be supposed from what has been already stated, that calculi of slow growth are firm and hard, and of high specific gravity; whilst those of rapid increase present precisely opposite qualities. The comparison, therefore, of the duration of symptoms with the indications of size supplied by sounding, is useful in leading to an estimate of the composition of the stone.

Mr. Crosse again dwells on the necessity of studying the nuclei of urinous concretions, and he adds a few illustrations of the subject.

1. A calculus may receive a sudden increase by the coalition of two smaller ones; about these a deposit takes place, so as to constitute a double nucleus. He has a sample of this in his possession ; it was removed from a girl by cystotomy.

2. The regularity or otherwise of the accretion is dependent on some circumstances, to which Mr. C. thus alludes. When regular layers, whether lithic, or composed of oxalate of lime, or of alternating layers of the phosphates, surround the central nucleus, he conceives that the stone was moveable in the bladder. For if, upon the contrary, the stone rests in one position, the deposition is not effected to the same extent on the surface which rests against the bladder, and the nucleus, of course, is no longer central. Mr. Crosse has two specimens of calculi illustrative of this occurrence. One was taken from a patient who, for a long time before death, was confined upon his back in bed, and the flat oval stone, formed in the bladder subsequent to stricture of the urethra, rested constantly on one of its sides, leaving the other exposed to the urine; the weight of the stone was sufficient to keep it in contact with the bladder, and the fresh accretion took place principally upon the free surface, which was rough and more convex than the other; the nucleus was of course not in the centre. The other instance was also taken from the bladder of a patient who was long recumbent on his back before death; the inferior surface of the calculus, on which it rested in contact with the bladder, was smooth, and the nucleus situated near to it. No one can doubt, from the appearances of a section of this calculus, and the smoothness of the surface near which the nucleus is found, that the stone had a fixed position in the bladder, always resting upon the part placed lowest in the figure, and that the fresh accretion, composed of the fusible calculus with much animal matter, took place upon the surface open to the urine. Mr. Crosse intreats the attention of pathologists to this interesting point.

In the succeeding case, the stone is supposed to have not merely reclined in one position in the bladder, but to have been grasped by its muscular fibres.

"A patient, in whose bladder a stone had been distinctly felt, assented to undergo cystotomy for its removal; but when a very valued friend of mine* went to a distance to perform the operation, no stone could be found on sounding; the disease was notwithstanding present, and the patient was worn down by his sufferings, after a year or two more had elapsed. Two calculi were found in his bladder; the smaller one, composed principally of lithic acid, and weighing two scruples was loose, and presented a regular shape. The larger stone has a nucleus of lithic acid, of a similar shape to the smaller moveable stone, which nucleus is situated so as to project upon the surface, whilst a very bulky deposit of the fusible kind, mixed with much animal matter, has taken place in one direction, and not all around the nucleus. To afford an explanation of such a partial deposit, we must suppose, that the small flat calculus of dark colour became caught and firmly held by the muscular fibres of the bladder, which organ, being in a very morbid state, the fusible calculus was quickly deposited upon the surface of the nucleus exposed to the urine." 14.

We need not pursue the subject farther; it follows from the preceding facts, that, supposing their explanation to be accurate, if a stone is found by section to have increased in a particular direction, it was not fixed or adherent to the bladder at that point. Mr. Crosse employs the terms fixed or adherent, for they are far from representing the same condition. Stones, he well observes, may be fixed by extending into the urethra or ureters, or by being grasped by the muscular fibres of the bladder, or contained partially in a sacculus, and nevertheless be in no degree adherent; indeed his investigations into this part of pathology have never shewn him a calculus actually adherent to the coats of the bladder, except by a layer of intervening lymph, soft enough to be easily broken through. He is, therefore, tempted to conclude that the numerous instances we read of, where calculi are said to have been found adherent in the operation, accounting for the difficulty in the extraction, have either presented nothing uncommon, or the calculus has been fixed in one of the several ways stated, and not actually adhered

* The late Dr. Rigby.

to the lining of the bladder. We fancy that most experienced surgeons will be found to agree with Mr. Crosse in this opinion.

### OF CALCULI IN THE KIDNEYS AND URETERS, AND THEIR PATHOLOGICAL EFFECTS.

Such is the subject discussed in the fourth chapter. Mr. Crosse alludes in succession to calculous matter contained in the tubuli uriniferi—to the anatomical disposition of the infundibula and pelvis of the ureter, and to calculi contained in them—to absorption of the secreting substance of the kidney—to the symptoms which characterize obstruction of the ureter—and, finally, to the modes in which these morbid states occasion death. We shall not pursue our author through his somewhat devious track, but shall alter the arrangement without interfering with the matter of his observations.

1. Gravel or small crystals of lithic acid have been said to be not unfrequently met with in the tubuli uriniferi of the kidney. Yet Mr. Crosse has been enabled in two or three instances only to find red gravel in this situation; and he believes he offers an unique case of the discovery of the oxalate of lime in the tubuli.

*Case.* An elderly man was treated for rheumatism and lumbago in a public institution, where he lost appetite, had a diminished secretion of urine, a parched brown tongue, and died in a few weeks. A calculus was found in the left ureter, seven inches from the kidney, completely preventing the passage of urine through that channel into the bladder, and inducing enlargement of the ureter above it, as well as of the pelvic cavity, which was filled with fetid, muco-purulent fluid; the lining membrane of this cavity was thickened and morbidly vascular. The right kidney was of small size, but normal in exterior shape, and in the condition of its pelvic and infundibular cavities. The parenchyma being cut through in different directions, the tubular part was found occupied by numerous white concretions, varying from the size of the smallest seeds to that of a large pin's head; these bodies were distributed over all parts of the substance of the kidney except the cortical portion. On minute investigation, Mr. Crosse found them to be pure oxalate of lime, crystallized, transparent, and situated in the tubuli uriniferi.

Morand has remarked that the rat is subject to calculi, and a neighbour of our author's has several times found pure oxalate of lime in the bladder of this animal. Mr. Crosse seizes the opportunity of remarking on a frequent error of pathologists, the association in the mind, of a calculus of oxalate of lime with the configuration of a mulberry. This shape is occasional, and usually assumed when the stone is of some size. The hemp-seed calculi, the most smooth and polished of all, are composed of oxalate of lime, which, when purest, is a white, crystallized, and transparent mass.

2. In order fully to appreciate the causes of the lodgement of calculous matter in the infundibula, perhaps it may be well to notice the following anatomical facts. The tubular substance of the kidney consists, as is well known, of a number of conical bundles of tubuli, which unite to constitute the mammillary processes. These are from twelve to eighteen in number, and their apices project into the pelvis of the ureter. Yet they do not open into the pelvis directly, but, sometimes singly, sometimes in a combination of two or three, into processes of the pelvis termed the infundibula or calyces. Now the point which must interest the pathologist is this. The infundibula are largest at the mammillary processes, and often contract, so as not to be one-eighth of an inch in diameter prior to their termination in the pelvis, naturally a cavity of small extent. From this of course it follows, that a stone of larger size would lodge in the upper portion of the infundibulum, than would readily pass through its contracted neck into the pelvis.

The membrane lining the pelvis, ureter, and infundibula, and covering the mammillary processes, in which it leaves openings answering to the orifices of the tubuli, is dilatable in a high degree. Sometimes the pelvic and infundibular cavities are found expanded into one large bag, in which there is scarce a remaining trace of the original form.

Mr. Crosse has met with several instances, and he mentions one, in which calculi formed, and increased to a considerable size in the largest part of the infundibula. In the case which he details the calculus was in contact with one of the mammillary processes, which had been partly absorbed to make room for its increase. Such are the facts connected with the lodgement of calculi in the infundibula. Our author indulges in some faint speculation on the symptoms which may indicate the passage of a stone into the pelvic cavity.

"Seeing," says he, "the narrowness of some of the infundibula before they open into the pelvic cavity, and that calculi do form and increase to a considerable size, (as I have found in other instances besides the preceding,) in the largest part of the infundibula, next the mammary processes, or what has been called the calyx, it appears to me that a nephritic attack, with violent pain, vomiting, and such other symptoms as attend the passage of a calculus along the ureters, may occur from its passing through the narrow part of one infundibula to reach the pelvic cavity. In a patient who suffered from nephritic attacks, the pain occupying the loins and not extending along the ureters, I found on dissection numerous small calculi in the calyces, some in the pelvis, and the infundibula so narrow at one part, that the calculi in the latter situation must have passed with difficulty from the calix. In a nephritic attack of this sort, one infundibular passage only out of several is obstructed, and the excretion of urine may continue, the others offering a passage for it into the pelvis: still we do not find a calculus fixed in the narrow part of an infundibulum, which I suppose is owing to the cavity so readily enlarging, and allowing the foreign body to reach the pelvis, where we most usually meet with it." 19.

The idea may be correct, the suggestion should be borne in mind, yet the cautious critic may exercise his vocation, and doubt if it be yet possible to distinguish the symptoms, which indicate the passage of a calculous concretion from the infundibula to the pelvis of the ureter. It is certain, and Mr. Crosse brings forward some instances himself, that the ureter or the pelvis of the kidney may be filled and obstructed with a calculus, nay, the substance of the kidney may be gradually absorbed, without the concurrence of prominent symptoms. There are few persons conversant with morbid anatomy who have not witnessed cases of this description.

3. It might probably be supposed that obstruction of the ureter would give rise to dilatation of the urinary reservoir above it, and perhaps to absorption of the substance of the secreting organ. The conjecture of reason is true in fact, and the proofs are of frequent and daily occurrence. The parenchymatous texture of the kidney may be absorbed in consequence of retention of the urinary secretion, whether that be the result of calculus in the bladder, enlarged prostate, or stricture, or obstruction of the ureter—however, in short, it be occasioned. Such absorption is generally attended with augmentation in size of the organ; but when it is produced by a deficient supply of arterial blood, or where the arteries are obstructed or diseased, a diminution of the volume of the gland ensues. In a man who presented one kidney healthy but enlarged, the other was reduced to a small bag; this atrophy appeared to be dependent on extreme ossification of the emulgent artery. We think we need not cite any instances of absorption of the glandular substance of the kidney, in consequence of obstruction of the ureter.

It is a curious question to inquire what quantum of glandular structure is sufficient for the purpose of secreting urine. A very thin remaining layer appears to be adequate to such an office. When one kidney is crippled or disabled in its functions, the other does double duty and commonly acquires additional dimensions.

Mr. Crosse observes that one ureter may be completely obstructed and the function of the kidney connected with it destroyed, without danger to the patient, or other inconvenience than pain; but if the other ureter become obstructed in like manner, the patient inevitably dies in six or seven days.

4. The symptoms produced by renal calculi are deserving of attentive study. Mr. Crosse's remarks are judicious and valuable.

"The evils produced by renal calculi are not to be measured by their size; a small passable calculus, in its course along the ureter, causes most severe and acute symptoms, which every practitioner is familiar with; a calculus firmly impacted in and filling the ureter, sometimes gives little pain, although leading to complete destruction of the renal organ; whilst a large calculus occupying the pelvis, and allowing the urine, as it is secreted, to pass on towards the bladder, with much pain brings no immediate danger, and may remain for many years, increasing sometimes to so great a size, that the parenchyma is absorbed to make room for it; and when the calculus is not very large, by keeping up irritation and an increased flow of arterial blood to the organ, it often leads to an augmentation of its glandular substance. A considerable calculus in the pelvis brings less danger of obstruction to the passage of the urine, than of acute inflammation, which arising in one kidney, often affects both and creates fatal suppression of the urinary excretion; in the absence of acute inflammation, a calculus in the pelvis creates a dull heavy pain in the loins, extending sometimes to the scapulæ, at others over the buttocks or to the groins and scrotum, and along the thighs; bloody urine after exercise; dysury and frequent micturition as if there were a stone in the bladder; and by chronic inflammation of the membrane lining the cavities of the kidney, an abundant catarrhal or muco-purulent secretion is formed, which appears with the evacuated urine.

In general one kidney is principally affected, the other being nearly or quite healthy; and we often find that when the urine is clear the patient has most pain, and expresses himself to feel easy when there is a plentiful mucous secretion with the urine; in the former case, the ureter of the affected kidney is temporarily obstructed and clear urine from the other organ, which is healthy, alone reaches the bladder. This state of ease when the urine is turbid, and increased pain when it flows clear, often attends disease of one kidney, unconnected with a calculus; but a marked succession of symptoms after the above order, always points out one kidney to be diseased and its ureter occasionally obstructed." 22.

An interesting case is detailed by Mr. Crosse. It is that of a man about fifty years of age, who had often passed calculous concretions composed of lithic acid. He had for some weeks complained of pain in the region of the left kidney, with frequent and painful micturition and turbid urine. The latter became clear, pale, and scanty, and suddenly urgent symptoms of a typhoid description supervened. The principal pain was in the site of the left kidney from whence it extended along the course of the ureter to the bladder. Only twelve ounces of clear urine were voided in twenty-four hours. The symptoms became aggravated—the urinary secretion ceased—coma, with stertor and dilated pupils supervened, and in eight or nine days from the commencement of the attack he expired. The disease was confined to the left kidney, which was buried in a great quantity of adipose substance, and was of large size, having the lining membrane of pelvis much inflamed; whilst a calculus completely obstructed the ureter, two inches from its commencement. The other kidney was much smaller, and apparently free from disease; its secretion had probably been suppressed from sympathy with the diseased one. The head was not examined, but no doubt effusion had occurred in it. And all these evils, as Mr. C. observes, were occasioned by a calculus escaping from the pelvis, and suddenly obstructing the ureter.

In another case, a small calculus in the ureter gave rise to suppuration and gangrene about the kidney. The patient was sixty years of age, and had suffered for some months from what was considered rheumatism in the loins, thighs, and knees. Twice after riding on horseback he was seized with urgent pain in the right kidney, attended by vomiting, and proving so severe as to cause him to scream out; the second of these attacks was accompanied by obstinate constipation; with a scanty evacuation of very turbid urine. Various remedies were employed. The urine soon became clear, but only half an ounce was voided at a time, and not above six ounces in the twenty-four hours—there were pain and tenderness on pressure in the region of the right kidney—the abdomen was prominent—the pulse 60. Spontaneous diarrhœa soon supervened—the pulse intermitted—the animal heat failed in a remarkable degree—and in six days from the commencement of the symptoms the patient died, the intellect remaining unaffected to the last.

Mr. Crosse concludes the chapter by a few observations on the rarity of ulceration of the mucous membrane of the kidney, and on the course which abscesses connected with the organ may pursue.

"I have been surprised to find how rarely, under extreme disease and profuse muco-purulent excretion, is the lining membrane of the pelvis and infundibula ulcerated; such a state occurs from scrofulous disease of the kidney, but rarely as a consequence of urinary concretions; when from their presence abscesses arise, these are situated in the surrounding adipose substance, and may burst into the pelvic cavity of the kidney, or into the peritonæal cavity, or into the contiguous part of the colon, or may make their way circuitously to the surface of the loins; sometimes the matter, besides bursting in one of the two last directions, also forms an opening into the pelvic cavity of the kidney, from which calculi may escape into the colon or outwardly upon the loins; numerous instances of the latter state of disease have been related; rendering it superfluous to pursue the subject by fresh cases of the kind." 25.

On glancing back at the chapter we now quit, we cannot refrain from remarking that it contains many interesting facts, and is worthy the perusal of the student and the surgeon. And here we may take the opportunity of indulging in one observation. In reviewing a work of this description, a work essentially composed of facts, yet enriched by a course of laborious reading commenced and conducted for the special purpose, criticism is equally uncalled for and absurd. The critic in such circumstances usefully sinks into the analyst, and the public require not the desultory, perhaps the hasty opinions of one who has paid little or no attention to the subject, but the mass of information collected with care by the author. Criticism is more adapted to works in which speculation predominates, to the keen examination of new views, the detection of fallacy, and exposure of ignorance. Few speak more plainly or more boldly than ourselves, few criticize more honestly, though many, no doubt, can do so more ably, when the proper occasions present themselves. But the aim of the conductors of this Journal is rather to disseminate useful information, and to foster the taste for fact and for induction, than to affect to say smart and clever things at the expense of the author, too frequently at that of truth. Our science is essentially a deep and a sedate one, and its philosophy is compromised by the indulgence of flippancy and levity.

### ON CALCULI IN THE URETHRA AND IN THE PROSTATE GLAND.

This chapter contains many facts connected with the subjects upon which it treats. Mr. Crosse successively alludes to calculi impacted in the prostatic portion of the urethra—in its membranous part—and in the substance of the prostate gland. To these varieties we shall successively allude.

A calculus which has just quitted the bladder not unfrequently becomes fixed in the commencement of the urethra, and gradually increases to a considerable magnitude, so as to lodge partly in the prostatic part of the urethra, and partly in the bladder. The following are the symptoms laid down by Mr. Crosse.

"A stone thus placed creates great pain, and is usually accompanied by constant stillicidium; it is easily felt with the sound, but this instrument meets with great obstruction when an attempt is made to introduce it into the bladder; indeed, if there have long been stillicidium, this viscus becomes so much contracted, that there is hardly a vesical cavity remaining to receive the end of the sound. The surgeon may recognize such a position of the stone not alone by the symptoms enumerated, but by the sound coming in contact with it before being passed deep enough to enter the bladder; and if the stone occupying the prostatic urethra be large, it can be felt by the finger introduced *per anum*. It is highly expedient that an operator should know when a stone occupies this peculiar situation, as he will meet with great embarrassment from proceeding, in such a case, under the idea that he is about to remove a loose stone from the cavity of the bladder." 26.

In illustration of this caution, Mr. Crosse relates a case. A very experienced lithotomist refused to operate upon a patient aged sixty-six years, on account of the bad state of his health, and the morbid condition of his bladder as indicated by the abundant muco-purulent discharge voided with the urine, but without detecting or suspecting any peculiarity in the situation of the stone. After this advice was given, the patient lingered in great misery between two and three years. Mr. Crosse had an opportunity of examining his bladder. A large calculus occupied its neck and the prostatic part of the urethra which were become one uniform cavity, the prostate gland being absorbed, and the fundus of the bladder contracted so as to present a cavity not bigger than a small nut-shell. The ureters and vasa deferentia opened into the cavity occupied by the stone.

Had an examination been made during life *per anum*, the stone would have been detected by the finger just within the sphincter. For the removal of a calculus so situated, Mr. Crosse recommends the lateral incision in the perineum, where a staff can be introduced into the bladder, or the operation of cutting on the gripe where it cannot. An instructive case is related by our author.

*Case.* "A lad, aged eighteen years, had suffered symptoms of stone in the bladder for more than half his life-time, and when a surgeon sounded him with a view to cystotomy, he found some peculiar circumstances, the sound meeting the stone early, and when fully introduced not striking fairly against it in the bladder, as is usual. For several years there had been stillicidium urinæ. Another surgeon of greater experience sounded the patient and said the bladder was filled with a large calculus, for the sound touched it in every direction; this was indeed the fact, but the inference as to a large calculus filling the bladder was wrong. Previous to an operation being undertaken, I had the opportunity of examining the patient, and found that the sound, on being introduced, came very soon in contact with the stone, before it could have gone deep enough to reach the bladder. With the finger *in ano*, I could feel the stone, as big as a pigeon's egg, through the coats of the rectum, immediately within the verge of the anus, and no prostate gland could be detected. The sound having passed behind the stone, I could feel it readily, with my finger *in ano*. Examining still more minutely by passing my finger along the perinæum, verge of the anus, and anterior wall of the rectum, till it had arrived in the bowel beyond the stone, I found no prostate gland, a proof that the stone was not in the bladder, but partly or entirely in the urethra. If any prostate gland remained, it was situated higher up than my finger would reach, and beyond the stone; in all probability this gland had become absorbed from pressure of the stone and obliteration of its ducts." 28.

In the operation, the gorget would not pass on, although the stone was not situated at a greater depth than an inch and a half from the external wound. The forceps would neither pass into the bladder, nor could their blades be opened so as to grasp the stone. After many rough attempts, the stone was turned out with the scoop; it weighed six drachms.

"I thought that in such a case, if the curved staff be used at all, it should be passed on the pubic or anterior aspect of the stone; if it be passed posteriorly or on the rectal aspect, there is great danger of wounding the rectum. By a free external incision, carried down to the stone, in the usual situation of the lateral operation, and by the scoop passed in on the pubic side, with the finger *in ano*, the surgeon may, if well acquainted with the situation of the stone, and assured of its not extending into the bladder, remove it with ease, expedition, and safety." 28.

To proceed with the sequel of the case. Some days after the operation the fæces passed through the perineal wound, proving the rectum to have been injured.* But in seven weeks the wound was healed, and the patient appeared cured. At the end of nine months he returned, with a calculus as large as a pigeon's egg in the urethra. The staff came in contact with the stone just beyond the bulb of the urethra, and passing behind it, entered the rectum by a fistulous opening. When the urine was voided, more passed by the rectum than by the penis, and when the bowels were relaxed, a little fæces got into the urethra, and was voided by the penis with the urine. This stone was removed by the lateral incision ; it weighed half an ounce. Yet the patient remained affected with recto-urethral fistula, and was again troubled with urinary concretions, several of which passed into the rectum and so escaped, the opening between the urethra and rectum being large enough to receive the end of the finger.

So much for calculus lodged in the prostatic part of the urethra and contiguous portion of the bladder. We have seen several cases of the kind. In one (the patient had stricture and false passage), the existence of a calculus was not suspected. In another many very able surgeons were deceived, although they examined the patient by the rectum. They were deceived, because they thought that the stone was of large size, so large as to preclude the ordinary operation of lithotomy. The recto-vesical operation was adopted, but the calculus proved of inconsiderable dimensions.

Mr. Crosse proceeds to the consideration of calculus occupying the prostatic or membranous part of the urethra, without extending into the neck of the bladder. It usually creates less urgent symptoms, and may remain there for many years without materially affecting the health. Mr. Crosse relates some cases in point. Sometimes, however, a stone in the membranous or prostatic part of the urethra will occasion very serious consequences. Mr. C. details a case in which the bladder was dilated, inflamed, and the seat of a gangrenous abscess at its fundus, while the ureters, and the pelvis, and infundibula of each kidney were enlarged. Yet, in this case, there was almost impervious stricture, and it is not quite fair to attribute the mischief solely to the calculus.

"When a calculus (says our author), of considerable size is situated in the membranous part of the urethra, immediately behind the bulb, if the finger *in ano* can be carried beyond it, so as to press it forward in the perinæum, it may be removed by a semilunar incision, as in cutting on the gripe; indeed if there be an insurmountable stricture anterior to the calculus, you can scarcely adopt any other operative proceeding." 30.

It is singular, as he observes, that calculi lodged in the urethra, even of large size, are sometimes not discovered with the sound or catheter, which it seems difficult to explain, unless we suppose that the operator, thinking only of the bladder, neglects the slighter impression conveyed by the stone in the urethra ; it may however happen that the muscles about the urethra, embracing firmly the instrument, prevent its touching the calculus,

* Wound of the rectum is far from unfrequent in the operation of lithotomy. Yet it seldom appears to be productive of mischief, and usually the wound in the bowel heals. We have seen several instances of this description.

which is generally lodged in a depression or cavity, formed by it and answering to its size. Calculi in the membranous part of the urethra, are sometimes discharged at the perinæum, by the processes of ulceration or of sloughing. In the instance of a lad of 17, a calculus weighing two ounces and a quarter, was voided in this manner through a fistulous opening in the perinæum. He lived for two years afterwards, voiding other small calculi by the wound, which never healed.

"Sometimes calculi in the urethra, quitting that passage by ulceration or otherwise, and getting into the cellular tissue under the integuments, descend into the scrotum, and still maintaining a channel of communication with the urethra, so that the urine gets access to them, they increase to an immense size; I am acquainted with a case where a calculus weighing eight ounces was removed from the scrotum of a man thirty-six years of age, and which I conjecture to have been formed in the above manner. The removal of such concretions is a very easy and safe operation; they are probably always composed externally of the phosphates, and if the explanation I give be correct, the cavity in which the stone is lodged will be found to communicate by a fistulous channel with some part of the urethra." 32.

In a note, Mr. Crosse refers to two facts:—one related by Gräfe in his Journal—the other by Valentine Mott. Gräfe's case was that of a shoemaker who had had for twenty years, a stone in the scrotum, which he supported in a bag; at length it got to such a bulk, that, during exertion at stool, it ruptured the scrotum and escaped: it weighed twenty-six ounces. The wound contracted and with surgical assistance healed. Mott's case was an instance of that kind of diseased action of the scrotum by which it increases, and becomes converted into a calcareous substance, independently of the urinary excretion. He successfully removed the disease by an operation.

Stones may lodge in any part of the urethra anterior to the membranous. But Mr. Crosse only deems it necessary to notice one case of this description. It conveys a hint which incautious practitioners should take.

*Case.* Mr. C. was summoned to a little boy, who had laboured for three days under incapacity of voiding his urine, accompanied by inflammation of the scrotum. This part was much swollen, and in a state of gangrene. On examination Mr. C. found a calculus lodged just within the orifice of the urethra, which it totally obstructed. He cut it out, and the urine flowed. But it was too late. The urethra had given way, the sloughing of the scrotum was the consequence of urinary infiltration, and in thirty-six hours the patient died. In the preparation which is preserved, the bursten part of the urethra answers to the anterior part of the scrotum; the bladder hangs flaccid, though its cavity is large; the ureter and pelvis of each kidney are greatly distended and increased, from pressure of the retained urine.

Our readers may be aware, that Sir Benjamin Brodie is more averse to cutting into the urethra for the removal of calculi anterior to the urethra than behind it. He thinks that urinary extravasation is more frequent after the former than the latter operation. We mention this opinion in connexion with and in extension of our author's observations, though, indeed, on this subject he may be said to offer none. We may mention that we lately saw an instance of this sort. A policeman applied to us on account of a pebble, which, he said, had got into his urethra. We could feel, both externally and with a probe, a foreign body in the fossa navicularis. As he said that it had entered, we concluded that it could be extracted. We seized it with a fine pair of forceps, and endeavoured to remove it in that manner. This could not be done, but after a little trouble we broke off some particles from the foreign substance, and readily extracted the remainder. It was a lithic calculus, and the policeman's story was, of course, untrue. A good deal of inflammation of the cellular tissue of the penis followed, but this after a time subsided.

Calculous concretions form in the ducts of the prostate gland. They are *not* deposited from the urine, nor derived from the kidney or the bladder; they are formed from the natural or disordered secretion of the gland, and uniformly composed of phosphate of lime.

Mr. Crosse has a preparation, and all surgeons must have witnessed instances, in which several small calculi, of the size of a pin's head, and of a brown colour, are observed in the substance of the gland, communicating with ducts that open at some distance on the surface of the urethra.

"It is only in this early stage that their true origin and situation, in the excretory ducts, can be demonstrated; for the urethral orifice of the ducts may close, or the cavities ulcerate, or enlarge as the concretion enlarges, until an extensive membranous cyst is formed, the substance of the gland being absorbed. When small and thus imbedded in the ducts, prostatic concretions cause none of the characteristic symptoms of urinary calculi and cannot be detected; it is only when they increase and rise so as to project at the orifices of the prostatic ducts, or escape, as they may do, into the urethra, that they can be discovered by the sound: but when of large size, or when numerous and contained in one cyst, they can be detected by the finger pressing upon the prostate gland through the rectum."*

It is when large, or numerous in one large cyst, or projecting into the urethra, that prostatic concretions give rise to the symptoms of stone, frequent, painful micturition, and discharge of mucus from inflammation of the urethra and neck of the bladder. Their frequent combination with stone, may lead us, Mr. C. thinks, to suspect that they are often connected as cause and

---

* "Concretions in the prostate gland, commencing in its ducts, often at a distance from their urethral orifice, even at the very bottom of a duct, go on increasing until each duct is enlarged into a pouch, rendering an escape of the concretion into the urethra, through the narrow orifice of the duct, impossible; the narrow orifice by which the pouch communicates with the urethra becomes often closed in consequence of inflammation and effusion of lymph; the pouch is a secreting cavity, which furnishes additional deposit; and as the concretions enlarge or multiply, the pouch enlarges in the direction where there is least resistance, towards the lateral or posterior surface of the prostate gland; thus, in extreme cases, the concretions are felt *per anum*, lying close to the rectum, and in a cavity no longer communicating with the urethra. The three plates of prostatic calculi form a series sufficient to support this explanation of their formation. Cases are on record of calculi passing away from the bladder by the rectum, (see *Memoirs of the Medical Society of London*, vol. iii, p. 536;) we can readily conceive how prostatic calculi, when large and in sacs no longer communicating with the urethra, may by abscess and ulceration take this, the shortest route, for their discharge." 34.

effect—the prostatic calculi being, in his opinion, the cause, the vesical the result.

The treatment briefly occupies Mr. Crosse. In extreme cases where a large prostatic calculus, or a cyst containing many small ones is discovered, it may be right, he conceives, to cut down to the prostate gland by the perinæum, and remove the concretions by the lateral operation of lithotomy. He quotes the sentiments and the practice of Mr. Wilson, Sir B. Brodie, and Sir A. Cooper. The first says, when prostatic calculi are very troublesome, and can be felt through the rectum, they may be cut out as in operating for stone by the gripe. Sir B. C. Brodie, in his Lectures on Lithotomy, says you may extract prostatic calculi with Weiss's forceps. Sir A. Cooper (*Surgical Lectures*) cut upon the staff, down to the prostate gland, and removed numerous calculi, which had not only excited painful feelings in the perinæum, but a degree of mental irritation bordering on insanity.

To recapitulate, prostatic calculi are not urinary concretions, but are formed, and may increase without the urine having access to them. They may notwithstanding rise to the orifices of the prostatic ducts, or get into, and be detained in the urethra, or pass retrograde into the bladder, becoming the nucleus around which a deposit from the urine takes place. It must also be remembered that prostatic calculi may, from the irritation they occasion, give rise to vesical, and that they may give rise also to all other alterations of the urethra, bladder and the kidneys, which follow on stricture and on stone, in short which are the formidable and familiar sequelæ of diseases of the outlet of the urinary apparatus.

With one other quotation devoted to another fact, we conclude the present chapter.

"Concretions of another sort about the neck of the bladder ought to be noticed. In aged persons, particularly with hypertrophy of the prostate gland, a bladder diseased, and the veins about it and about the rectum varicose, concretions of phosphate of lime, varying in size from a pin's head to a kidney-bean, are often found in the veins ; sometimes they present the appearance of a white pea, and an equality or projection is observable answering to the surface by which the body adhered to the coats of the vein. These concretions have no connexion with the urinary or any other excretions, and should not be regarded as calculi, they are a morbid growth from the coats of the vein, to which at an early period they are invariably adherent, and a membrane covers them, upon the surface where not adherent, which I presume is the extended inner coat of the vein, the morbid growth originating in the outer coat. Fig. 6, (a) of plate ii, shews a portion of vein containing one of these concretions, and d, e, f, g, exhibit them of different shape and size, their chemical composition is chiefly phosphate and carbonate of lime, and they approach nearer to ossification than to calculous concretions. I remember that Professor Meckel has well represented them, but know of no English author from whom they have received the same attention." 36.

### On Calculi in the Urinary Bladder, and their Pathological Effects.

From describing the nature of calculi in the bladder and their history, when placed in the urethra, Mr. Crosse proceeds to the enumeration of their pathological effects, on the organ that contains them, on the kidney, and the urethra. Yet his diffidence induces him to render the chapter incomplete and unsatisfactory, and he admits that the wish to advance scarcely more than he can demonstrate, has prevented him from rendering his statements so complete and so systematic as they might be. We respect the motive, but regret the result. The mass of the profession are still in a state of comparative ignorance of the chain of consequences, pathological consequences, that follow on diseases of the urethra and the bladder. A methodical investigation and an accurate account of them, would undoubtedly prove a valuable addition to the information generally possessed.

Our author judiciously avoids the enumeration of the symptoms occasioned by stone in the bladder. It would be well if other authors would waive with equal sense the repetition of the school-boy's lesson. He successively adverts to the pathological effects of vesical calculi on the bladder, the urethra, and the kidneys. For the reason we have stated, the catalogue is incomplete, and Mr. Crosse confines himself, or nearly does so, to what he has personally witnessed and can demonstrate. We may remark too that Mr. Crosse's style and method, are not calculated to give effect to the matter they display; for his style is inelegant, and his method is confused. We look in vain through the laborious page, for the ornament of the accomplished, or the clearness of the practised writer.

The effects on the bladder of a calculus in it, may simply be referred to inflammatory action, and to the efforts of the muscular substance of the organ, occasioned by its irritation. From the first results extreme vascularity of the lining membrane, an abundant muco-purulent discharge from its surface, rarely ulceration, but sometimes sloughing. From the second, arises excessive thickening of the muscular coat, or sacculi of the mucous membrane between its fibres.

Chronic inflammation of the mucous membrane of the bladder is a common consequence of the presence of a calculus. We have stated that ulceration is rare, indeed it is surprising how very rare it is considering the marked disposition to this action evinced by mucous membranes in other parts of the body. It might be expected from what we know of the œconomy, that the violent straining to repel the urine occasioned by the residence of a calculus in the bladder, should give rise to much thickening of its muscular coat. That thickening is observed, from a similar cause, in cases of long continued stricture of the urethra, and in cases of enlargement of the prostate. Whatever calls for augmentation of effort on the part of the bladder, tends to the production of this pathological condition. Abscess is not infrequent in the thickened coats. The matter may find an outlet into the cavity of the bladder, or it may escape into the peritonæal cavity and prove destructive; or without the abscess bursting, fatal peritonitis may be induced. These abscesses in the thickened coats of the bladder slough or become gangrenous in extreme cases, and aged persons, who have for years been sufferers from stone, usually die from this cause. There are also other morbid conditions determined by the demand for violent expulsive efforts:—such are the augmented power and size of the muscles about the neck of the bladder, levator ani, transversales and ejaculator—the general fulness of the perinæum—the varicose state of veins about the rectum and neck of the bladder,—consequences of painful dysury and vesical tenesmus.

The thickening of the bladder may actually proceed to such an extent, and may be accompanied with such contraction as to grasp the contained calculi, and not allow half an ounce of urine to remain in it. This happened in the case of Dr. ——, in whom an enlarged prostate gland was followed by four vesical calculi, which at death just filled the cavity and were so tightly compressed, that the lining membrane was indented by them, and still retains the impression in the preparation preserved by Mr. Crosse.

Sacculi of the bladder are the legitimate offspring of the same class of causes. During the forcible contraction of the organ, the mucous membrane is forced, by the re-action of the fluid, into the interstices between the fibres of the muscular coat. Such at least is the ordinary, and perhaps, in general, the correct explanation. It is certain that these sacculi are met with in a great proportion of persons who have laboured for a long time under stone. They are usually small, but when once formed, they may go on enlarging to a great size, and if numerous, may become capable of containing more urine than the proper cavity of the bladder. It is mostly in the adult and the aged, that sacculi of the inner membrane are met with. Mr. Crosse has never seen them in the very young. He believes that their occurrence would be more frequent, were not the bladder to thicken in its muscular coat and remain contracted close to the calculus; it is where the bladder is not contracted, and its coats not much thickened, that we find large sacs of the inner membrane, which cannot form when the coats are thickened and the cavity small.

"Although sacculi are so frequent, large sacs are rarely found, and, consequently, a sacculated calculus in the bladder, formerly so often spoken of, and now occasionally reported by an unsuccessful operator to be present, may be considered a rare occurrence; it is however one of the complications of this disease, which the surgeon ought to bear in mind, and have a clear notion of."* 38.

Before he dismisses the subject of sacculi, Mr. Crosse observes that they frequently arise from stricture of the urethra, independently of calculus. He notices a fact which has attracted the attention of other surgeons—the occasional simulation of the symptoms of stone when none is present. Mr. Guthrie,† who has lately drawn attention to this circumstance, attributes its occurrence to a sacculus, containing urine, coming down with a sort of blow upon the instrument; and he talks of the fluttering strokes of the bladder.

---

* "The late Mr. Martineau, in his paper on lithotomy in the *Medico-Chirurgical Transactions* (vol. xi. p. 404), has expressed doubts about calculi being sacculated, giving rise to difficulty in the operation, which shews the disadvantage of trusting to individual experience, without the aid of extensive reading and examination of pathological collections. This present month (October, 1834), in a hospital patient who had for twenty or thirty years been affected by stricture of the urethra, and died from perinæal abscess and urinary infiltration, arising from rupture of the urethra behind the stricture, I found a large sac, or hernia of the inner membrane, just above the termination of the left ureter, filled with a calculus of large size, which also projected into the cavity of the bladder, the entire calculus being of an hour-glass shape, with the isthmus answering to the narrow neck of the sac. A precisely similar specimen is preserved in the museum of the Royal College of Surgeons in London."

† Our readers may turn to our review of that gentleman's work.—Eds.

Mr. Crosse, less speculative, expresses the fact, but does not venture on an explanation. His case, for he offers one, is instructive.

*Case.* A gentleman had for many years severe stricture, which was at length completely and permanently cured; but painful, frequent micturition continued ; he was repeatedly sounded for stone and had the opinion of many surgeons of eminence, without obtaining entire relief; the urine deposited much mucus; after suffering thus several years, he died, and dissection threw no other light upon the cause of the painful symptoms than the numerous large sacs of the inner membrane.

We lately saw an instance in which it was presumed that sacculi existed. But it was presumed by a surgeon, who pronounced on the presence of a stricture that apparently existed only in his want of dexterity or conscience. We were consulted by a gentleman on account of a stricture, under which he said he laboured. He had been under the care of a surgeon of some little repute in London, who not only told him he had stricture of the urethra, but sacculi of the bladder also. He said that he had sacculi principally on account of the occasional violence with which the end of the instrument was struck, when introduced into the organ. We passed without difficulty a No. 14 sound into the bladder. That convinced us that no permanent stricture existed. We repeated the introduction of the sound with facility on two subsequent occasions. We were certain that there was no stricture—we were not sure that there were sacculi, for the urine was merely a little too acid, and the "fluttering strokes" were never felt by us. We prescribed those remedies that were calculated to improve the condition of the digestive organs, and to lessen the acidity of the urinary excretion, and we relieved the gentleman from much of the anxiety and alarm which he had laboured under. Our opinion was confirmed, and our practice sanctioned by Sir Benjamin Brodie, who, at our request, saw the patient.

Stricture of the urethra is more frequently a cause than a consequence of vesical calculus. But sometimes inflammation extends from the bladder to the urethra, and severe permanent stricture at the membranous part is produced. Sometimes the stricture arising from inflammation is suddenly established.

*Case.* A patient in advanced years suffered inflammation of the lining membrane of the bladder, apparently in consequence of numerous small concretions of lithic acid lodging in it, after descending from the kidney ; the inflammation extended to the urethra; there was complete retention of urine, and difficulty in introducing an instrument. A surgeon, in rude attempts with the catheter, made a false passage anterior to the prostate. The patient died.

It is easy to suppose, what is the fact, that the combination of urinary calculus with stricture is productive of the worst effects. In a case of this sort that occurred to Mr. Crosse, stricture gave rise to disease of the bladder, to stone, to fistulæ in perineo, and to death. On dissection, the bladder was found greatly thickened in its coats—its lining membrane covered with adherent lymph, and in some places gangrenous—the urethra behind the stricture much dilated, and broad bands extending across the passage at this part, forming a net-work, behind which a calculus, apparently of lithic acid, was detained.

The alterations effected in the kidneys must be a source of the highest interest to the modern pathologist and surgeon. We repeat that those changes are imperfectly understood by the best informed amongst us; perhaps not at all by the routine practitioner. Let us hear what Mr. Crosse has to advance.

"When an urinary vesical calculus has been formed for years, and has brought on severe symptoms, and especially if attended by stricture of the urethra, or enlarged prostate gland, the kidneys, if before healthy, become involved; the severe disury causes enlargement of the ureters from distention of the retained urine, and inflammation extends along them, even to the kidney itself. The pelvic cavities also become altered in shape and enlarged, the infundibula extended or unfolded, and the lining membrane of all the cavities thus acted upon, from repeated attacks of inflammation, is thickened and furnishes a catarrhal secretion. The parenchymatous substance of the kidney is more or less absorbed, the mammary projections are obliterated, spurious hydatids occupy the cortical part, and all the serious evils (ulcerations, contiguous abscess or gangrene) described in speaking of calculi in these organs, are met with as sequela of the vesical calculus." 41.

He subjoins two cases in illustration of this very brief, and we must say very imperfect, epitome of the morbid conditions of the kidney.

*Case* 1. In this patient, the bladder was much thickened and contracted, with stricture of the urethra, and an enlarged prostate gland. One kidney was but little altered, the pelvis only being dilated. But the other was very large, and buried in an enormous mass of fat, in which abscesses were situated. The whole substance of this kidney was softened and parts of it gangrenous; the pelvic cavity much enlarged, filled with fetid urine and mucus, and its lining membrane thickened and studded with clots of extravasated blood.

*Case* 2. The stone, in this instance, was consecutive to an enlarged prostate gland. The latter seemed to occupy half the cavity of the bladder, by a general enlargement of the lateral lobes. The coats of the bladder itself were much thickened at the fundus, where an abscess had formed. One kidney was greatly diseased; the mammary processes were obliterated—the pelvis much enlarged—and its lining membrane thickened and extremely vascular.

In reference to this case, Mr. Crosse alludes to a practical point in the management of the catheter in cases of enlarged prostate, which we may incidentally touch on. Perhaps we had better let our author speak.

"I had frequently (says he) to introduce the catheter, during the two or three years that I was almost daily in attendance on him, and could only succeed by using the gum-elastic instrument; and when I had passed it down to the prostate gland, I held the stilet fast, and pushed the canula off it for two inches, by which manœuvre, the end of the canula, as it passed through the prostatic portion of the urethra, was turned upwards, answering to the curve of the passage, and thus entered the bladder; I could never succeed but by adopting this method."* 41.

* "The late Mr. Hey, in his *Practical Observations on Surgery* (p. 437, plate

### On Sounding for a Stone in the Bladder.

Mr. Crosse dedicates a separate chapter to sounding, and takes a comprehensive view of this, too often thought a trivial operation. The lithotritists have certainly done much to enhance its applicability and use, and the necessity which now exists for the detection of small stones has led to the employment of much care, and the accomplishment of some improvements in the method of proceeding. Mr. Crosse appears to think strongly on the subject.

" Few operations (he says) are more abused and less skilfully practised than this, an instrument being unceremoniously thrust into the bladder, wherever there are symptoms resembling those of stone; and not only the young and over-zealous, but even the well educated and more experienced surgeon, persists often in making repeated examinations to find a stone, where none happens to be present, fearing lest he should fail to detect it, and lose the opportunity of éclat from a case of lithotomy. I have observed two strong reasons against the practice of frequent and persevering sounding; first, because it is not without danger, and secondly, because there are so many other morbid conditions of the urinary organs, giving rise to symptoms resembling those which a vesical calculus ordinarily produces. In consequence of persevering and unsuccessful attempts to discover a stone with the sound, in a little boy, inflammation of the bladder came on, attended by vomiting, and extending to the peritonæum; the most active antiphlogistic treatment failed to arrest it, and death ensued in four days." 48.

Deserting the exact path pursued by Mr. Crosse, we shall first determine the most proper mode of performing the operation, and then take notice of some cases in which it may be fallacious or improper.

A solid instrument of steel is the best sound. It should be as long as a catheter, and curved for the last three or four inches, that it may project into the bladder; it should not be so large as to fill, much less to distend the urethra, but of moderate size, that it may readily be moved backwards and forwards in this passage, and its curved part turned in different directions in the bladder. Every surface should be well polished, and the handle, being always intended to suit the operator, should be broad enough to receive the thumb and two fingers, and not small, whatever be the size of the rest of the instrument. It is necessary to have the handle well polished, that the fingers may touch a greater surface, and receive and recognize the most delicate impression; by being wedge-shaped, or thinner at its extremity than next the body of the instrument, it has the advantage of increasing the impression conveyed to the touch by any resisting body, when the instrument is pushed onwards, the most usual and always the first movement to be given it in sounding. The name of the instrument-maker should *not* be on the handle, as it interrupts its necessary smoothness.

Children are never sounded in the erect posture; but the first examina-

---

ii. ed. 1810), first described the effect of withdrawing the stilet of a flexible catheter a little, increasing its curvature and bringing the end of the instrument forwards. I refer to a different manœuvre, holding the stilet fixed, and pushing the catheter off to the extent of an inch or more: one of these plans will often succeed, in cases of enlarged prostate, when the other fails; and often the surgeon accomplishes his object by adopting each method in succession."

tion of an adult should be made in that position. The sound should be introduced with the utmost possible gentleness and lightness, when a stone of any size, if loose in the bladder, usually falls down upon its neck, and is felt by the instrument on entering. The methodical exploration of the bladder is so well described by Mr. Crosse, that we would not mar nor mutilate its consistency. We give it to our readers entire.

"By alternately depressing between the thighs, and elevating the handle of the curved sound, you vary the extent to which it projects into the bladder, and will often in this movement feel a grating of the calculus against the instrument; the impression thus obtained is usually obscure and seldom to be relied upon. With the handle depressed more or less, and held centrally, answering to the median line of the body, you may jerk it upwards and backwards towards the rectum, when it will strike a stone lying in that direction, producing a sensible resistance and often also an audible sound, satisfactory evidences, when conjoined, of a calculus being present. You may give the same movement to *the sound,* with the handle inclined more or less to one groin, and thus explore each lateral, as well as the posterior part, of the bladder. Should a stone not be felt under these movements of the sound, you may suspect it to be on the pubic or concave side of the instrument, when it will be felt by your drawing the instrument downwards and forwards, which movement should be performed first with the handle answering to the median line and more or less depressed between the thighs, and afterwards, with it inclined obliquely to either side, which will explore the lateral and anterior part of the cavity; if in any of these trials, the stone be felt, touching the concave side of the sound, you know it to be situated towards the os pubis; you may also, with the curved sound projecting considerably into the bladder, turn the handle to some extent upon its own axis, making its extremity describe a part of a circle, and sweeping the upper and lateral parts of the bladder. By a practised hand, the sound is in a short time made to perform these different movements, and the object is, by a regular succession of them, to carry the sound to every part of the vesical cavity.

Where a careful sounding is required, the patient should be placed horizontally on his back; indeed the surgeon should always bear in mind how advantageous it is to vary the position of the patient, and how much may be gained by so doing. If, when the patient is standing, the stone be felt on the pubic side of the instrument, and when dorsally recumbent, you find the stone behind it, towards the rectum, you know it to be moveable and loose. The sound being in the bladder, the shoulders of the patient may be raised into the half-sitting posture, or they may be depressed greatly so as to have the pelvis on the top of an inclined plane and make the axis of the spine answer to an angle of forty-five degrees, this latter being the method to remove the stone from the neck of the bladder and carry it to the fundus: he may likewise be placed on either side, or upon his hands and knees with the face downwards; all these changes of position should be made after the sound is introduced; the last is particularly applicable to cases of enlarged prostate gland, behind which there is a cavity not accessible to the long curved sound by any movement that can be given to it; and you can only remove this defect by changing the situation of the stone in the bladder, which is accomplished by altering the position of the patient.

Where the sound touches the stone in different directions, and is found to pass over a large surface of it, you may conclude it is of large dimensions; but when, under the same position of the body, you do not feel it repeatedly, on passing the sound to the same part of the vesical cavity, it is likely to be small." 53.

The quotation is a long one, but the matter is too valuable to make us regret its transference to our pages. The young surgeon, and perhaps the old one, may usefully peruse the precepts of our author.

Mr. Crosse recommends the employment of auscultation as an useful assistant to the sound. He thinks that it is susceptible of much improvement, and likely to be turned to a good account.

The bladder requires to be examined when empty as well as when full, and during the act of voiding its contents an instrument introduced into it will sometimes detect a stone, incapable of being felt upon other occasions. Sir Everard Home was in the habit of recommending the use of the gum catheter, but Mr. Crosse appears to be averse to it, and prefers the instrument of silver. It should be used as a sound before the urine is drawn off, and again in the same manner after its evacuation. The catheter is useful also in determining whether the bladder is capable of emptying itself completely. And it is so important for the surgeon to know how much urine the organ can contain, and whether what it does contain can be totally expelled, that the use of the catheter is on this account desirable.

If the surgeon intends to operate with the straight staff, he must introduce previously a straight sound; but such an instrument can be expected only to detect a moveable stone resting at the neck, posterior part or fundus of the bladder, and is not suited for exploring the whole of the vesical cavity, nor capable of taking a great variety of movements.

Baron Heurteloup would seem to be the first who suggested what may be denominated *rotatory sounding;* effected by a sound with a very short curve, the part in the urethra being straight. Such a sound admits of being turned round upon its axis, when its curved end will describe a circle in the bladder, and pass behind the prostate gland, searching the *bas-fond* of this organ, which is just the part of its cavity least accessible, sometimes quite inaccessible, to the long curved sound. Mr. Crosse, however, repeats the natural and often urged objection, that neither a sound of this description nor a straight one is adapted for all cases, certainly not for those in which the prostate is enlarged. He is convinced that he has known serious and even fatal injury inflicted on this gland, by the lithotritist in his attempts to introduce a straight instrument into the bladder under such circumstances.

Mr. Crosse dwells forcibly on the paramount necessity of examination per anum, before the operation of lithotomy is undertaken.

"In young patients," he observes, "we can feel through the rectum the whole outline of the bladder, and often tell the size and situation of the stone contained in it. In the adult, examining by the rectum enables you to detect a calculus in the membranous or prostatic portion of the urethra, or acquaints you with the size and condition of the prostate gland; and when the finger is long enough, you can tell the state of the bladder, as to tenderness on pressure, and thickness of its coats. Not unfrequently you can feel the stone by the rectum, balance its weight upon the finger, or tell its shape, its surface and situation. A good deal of tact is however required to profit by this mode of examining; in a child during violent screaming and straining, the coats of the bladder contracted so firmly on the contained urine, that it feels a tense body, shaped like a walnut; and no stone can be detected, till this state has subsided. In judging of the size of the stone, you must allow for the thickening of intervening parts, or when the bladder is much thickened you will estimate the stone to be larger than it really is. When the bladder contains a few ounces of urine, and the stone is not of considerable size, it gives so evasive an impression to the finger *in ano,* that much practice is required to profit by this examination and to tell that you feel a stone; but where this foreign body is very large, it can always be felt by the rectum, unless there be a greatly enlarged prostate gland or other tumor interfering. In this method of exploring the bladder, the patient is placed horizontally on the back, or with the shoulders raised; but the surgeon has more command, and sometimes facilitates his object, by making the patient stand on a bed or table; if the bladder be empty, only gentle pressure with the finger should be made, as it will injure the lining membrane, particularly if the calculus be rough on the surface. I have known so much mischief arise from the undertaking of operations for stone without previously examining by the rectum, that I am induced to represent forcibly the propriety of doing it in every instance; I should deem myself as little justified in omitting this method of exploration, as in operating without having sounded at all." 55.

Such then are the precepts laid down by Mr. Crosse for exploring the bladder with the sound. Reversing his arrangement which is seldon very clear, and slightly condensing his remarks which, though free from diffuseness, are not couched in the simple and elegant style of one accustomed to literary composition, we may now turn our attention to some of the circumstances which interfere with the operation of sounding—sometimes requiring its execution to be delicate, sometimes rendering its indications false. We say to some of the circumstances of this description, for Mr. Crosse certainly does not mention all. But we have limited our task to that of analysis, and shall therefore do no more than follow our author. He confines himself to alluding to the presence of tumors in the bladder.

When these are present, particularly when of a vascular or malignant character, sounding is very deleterious; and such cases are attended by symptoms very like those of stone. In a patient whose bladder is represented in one of Mr. Crosse's plates, and where a vascular tumor, planted round the termination of the right ureter, projects extensively into the bladder, many soundings were practised, and always followed by great bleeding and an increase of the patient's sufferings. He lately had under his care a tall middle-aged man, suffering from such symptoms as a stone in the bladder would produce; he had become much emaciated in a few months, and almost daily lost some blood from the bladder, even when quiet and recumbent, and particularly after the use of the sound. Mr. Crosse was satisfied with three gentle and guarded examinations. He could detect no stone, and attributed the symptoms to a vascular tumor in the bladder.

Mr. Crosse occupies five pages with a case, the details of which he thinks it would be inexcusable to omit. The case itself is highly interesting, but a very brief space will permit us to present its principal features.

*Case.* Master C. was about one year and a half old when Mr. Crosse was first consulted respecting him. He had the symptoms of calculus in the badder in a severe degree. Every two or three weeks Mr. Crosse gently employed the sound, and twice, he thought, but he was not satisfied, that he felt a stone; this was on each occasion on passing the sound towards the left side of the bladder. In consultation, another surgeon thought he felt a calculus. The child was sinking under the severity of his sufferings, and at the age of two years Mr. Crosse performed the operation of lithotomy. What happened he himself shall tell.

"The same experienced surgeon who had met me before in consultation, now gave his assistance, and stated again his opinion that he felt a stone; I stated I could not do so, although I introduced the sound, as well as the staff; but I felt a resisting body at the left side of the bladder, about the termination of the left ureter. I hesitated about proceeding further; after a few minutes delay, however, I determined to cut into the bladder, and re-introduced the staff for this purpose. I observed there was a great fulness of the perinæum; as soon as I cut down to the staff, and opened the membranous part of the urethra, a semi-transparent substance appeared in the wound, resembling the mucus which had been passed from the rectum by the child's straining when first placed upon the table, and I feared that I had, for the first time in my life, wounded the rectum! and the same impression, I afterwards learnt, momentarily pervaded the minds of the numerous bystanders. This accident was not, however, sufficient to baffle me, nor to prevent my proceeding in the rest of the operation in which I was embarked. With the assistance of my left fore-finger, guided by the staff, I carried the scalpel forward fairly to the neck of the bladder, and on withdrawing the knife, I observed that the wound became instantly filled with a mass resembling, on this sudden view, what one would have expected to see, had I opened the peritonæum and allowed the processus vermiformis and several folds of the small intestines to protrude; such was the momentary, frightful impression, that held the mute attention of myself and all who were present! I pushed back the protruded parts, carrying my left little finger into the bladder, where I could feel no stone, but found the cavity filled with soft tumors, with a firmer substance near the orifice of the left ureter. If I betrayed any judgment in the case, it was undoubtedly in this stage of the business, by relinquishing the forceps held in readiness for me, and withdrawing my finger from the bladder. The same parts then protruded, as on the bladder being first opened, and they proved on inspection to be tumors, connected together like a cluster of grapes, some more some less transparent, resembling in firmness, appearance and structure, the mild polypus nasi; the membrane by which they were connected with each other and with the inner surface of the bladder, was long and loose enough to allow some of the tumors to hang externally dependent at the wound, and I have no doubt that, by the violent straining efforts of the child, when first placed on the table, they had entered the urethra; this is indeed proved to have happened, by the tumors appearing in the wound the instant I bared the staff, and it accounts for the fulness of the perinæum which I noticed immediately before commencing the operation." 46.

The similarity of these tumors to the simple nasal polypi was evident, and Mr. Crosse endeavoured to remove them gently with scissors. But much remained in the bladder that he could not cut away. Great irritability of the bladder succeeded, the straining efforts were perpetual, and in forty-four hours after the operation the patient sank.

On examination after death, the bladder was found much thickened—at its fundus, there was a convex prominence covered by peritoneum, which, when cut into, was found to be a firm mass of thickened cellular substance, situated external to the muscular coat, and containing a small central cavity. The fatal disease, however, occupied the lining membrane of the bladder, which was loosely connected with the muscular coat, and very abundant so as to fall into folds, also thicker than usual, and having a gelatinous appearance. The cavity of the bladder was still occupied by tumors, growing from the lining membrane, and situated at the inferior part near its neck. One large tumor, with a broad basis, was firmer than the rest, and placed near the termination of the left ureter; this must have been the resisting body so generally felt on sounding. Several small detached tumors, from the size of a pea to that of a bean, were loose in the bladder. Towards the neck of the bladder the tumors had a different structure, and presented a wart-like surface; but all the tumors were covered with their proper membrane, continuous with the inner coat of the bladder, which was uninjured except in three or four spots, where Mr. Crosse had cut off the tumors with scissors. The neck of the bladder and prostatic portion of the urethra were much dilated, and the narrow basis, by which the tumors about the neck of the bladder hung, was sufficiently loose to allow them to descend into the prostatic and membranous parts of the urethra, which no doubt happened during life, causing the fulness of perinæum, and accounting for the foremost of the tumors prolapsing through the wound, as soon as the urethra was opened behind the bulb. The disease was exclusively seated in the mucous membrane, none of which was in a healthy condition, being loose, gelatinous, and thickened in all parts where polypous tumors did not arise, from the termination of the ureters to the fundus.

Mr. Crosse remarks that were it possible to ascertain beforehand the existence of *simple* polypous tumors in the bladder, an operation *might* usefully be performed for their removal. But he also owns that the diagnosis is attended with great difficulty, and he almost doubts the possibility of its being made with sufficient certainty to justify the surgeon in resorting to the extreme measure. On this subject he has collected the opinions and the practice of many surgeons which he embodies in a note. The note is not unworthy of attention, and we shall introduce it here.

" Callisen says," writes Mr. Crosse, " in reference to the *polypus vesicæ,* that the catheter, injections, &c. must be used,—' si vero, quod sæpissimè accidit, his resistat malum, ad incisionem colli vesicæ tumorisque sequentem irritationem, macerationem, ligaturam, evulsionem, prout rei indoles suaserit, indicatam recurrendum erit; quæ omnia sub ischuriæ et calculi vesicalis chirurgia uberius exponentur.' (*Systema Chirurgiæ Hodiernæ,* tom. ii. p. 190.) Le Cat invented an instrument for extirpating fungous excrescences of the bladder. (*Philosophical Transactions,* vol. xlvii. p. 29.) M. A. Petit of Lyons performed cystotomy, on a young woman, supposing there was a stone, other persons believing they felt one as well as himself; but it proved to be a polypous tumour of the bladder, and they agreed that nothing could be done; the patient lived a year afterwards, and Petit, on dissection, ' trouva dans la vessie, un polype du volume du poing, d'une forme pyramidale, et tenant par un pedicule extrêmement étroit.' The narrator of this case (*Dictionnaire des Sciences Médicales, Article Polype,* tom. xliv. p. 233) says, that under such circumstances, with a polypus having a narrow neck, you should tie and extract it; but in every other case, he advises not to lithotomize, even though it be thought that a polypus is felt on sounding. Lassus (*Pathologie Chirurgicale,* tom. i. p. 522) has some good remarks on fungous tumours of the bladder, which he likens to polypi of the pituitary membrane, having a pedicle; but I suspect he has confounded with them tumours of the prostate gland, projecting into the bladder. Covillard (*Obs. Iatrochir.* p. 93) performed cystotomy for a tumour in the bladder, the size of a nut; ' je la mouchait avec les tenettes, ce que réussit de sorte qu'en moins de huit ou dix jours, la dite tumeur termina par suppuration;' and in a month the patient was convalescent. The most remarkable case of a tumour, growing from the inside of the bladder, and successfully extirpated, is related by Warner.—(*Philosophical Transactions,* A. D. 1790.)" 49.

### ON REMOVING VESICAL CALCULI THROUGH THE URETHRA.

It is probable that one of the greatest improvements which modern times have effected in the treatment of calculous disorders, is the application of the forceps to the removal of small stones from the bladder. Chemistry, by teaching us the composition and morbid alterations of the urine, and the labours of the lithotritists, by giving precision to the examination of the bladder, have enabled us to detect the existence of calculi when still of inconsiderable size. When detected, they may almost always be removed by a surgeon of moderate dexterity. We would impress upon our readers, in the most emphatic manner, the propriety, the necessity, of directing their attention to the early symptoms of stone in the bladder. Then is the time for surgery to interfere with safety and effect—then is the time for science to achieve its highest triumph, in displaying, by the combination of manual skill and elaborate knowledge, the arrest of a most painful and formidable malady. We repeat, that the surgeon is ignorant of his art, or careless in its practice, if he suffers the incipient symptoms of stone to elapse without examination of the bladder—if he permits a small calculus, which might be extracted, to grow into a large one which cannot. Lithotrity may be a very great improvement, but the extraction of calculi by the urethra is a greater—greater, indeed, by the ratio that almost certain prevention bears to a most uncertain cure.

Mr. Crosse observes well, that although, from dilatation of the ureters, calculi may descend into the bladder which are too large to pass through the urethra, this is the rare exception, not the rule. In general it may be stated, and it ought not for a moment to be forgotten, that every stone in the urinary bladder must at one period have been so small, that it might have been removed through the urethra. To a conscientious and well-informed surgeon, this reflection must be calculated to exert a strong influence upon his mind.

The object of the chapter on which we have entered is twofold: to point out, with as much precision as possible, the symptoms denoting the presence of small stones; and to offer some suggestions on the best mode of removing them after they are discovered. And first, then, of the symptoms.

1. Perhaps the best indication that can be obtained, of a calculus in the bladder being of small size, is to have traced it, not long before, passing through the ureter from one of the kidneys; but this source of information is rarely afforded, and the surgeon must trust to, and form his judgment upon, the reported duration of the symptoms, the preceding and present degree of their intensity, and the evidence derived from sounding the bladder. That evidence is thus stated by the observation of Mr. Crosse.

"Where symptoms of stone have not existed above two or three months, or have been absent for a time and suddenly returned in a severe degree, producing itching at the end of the penis, frequent painful micturition, and occasional retention of urine, we may suspect that a small calculus is present in the bladder. With a small stone, the patient is often free from all inconvenience for a day or two, or even a week or two, and then is suddenly seized with retention of urine and most distressing pain, from the calculus entering the commencement of the urethra. In the interval between these sudden and acute attacks, the patient experiences only a slight itching at the end of the penis, irritation about the neck of the bladder, and a more frequent and sudden call to pass urine than is healthy.

On sounding at this early period, you will occasionally find such an audible click, or noise produced, when you strike the stone, as can be heard at a distance of several yards; and the evidence thus obtained, more audible than tangible, arising from a clear and sharp sound, I have experienced only when the stone is small." 58.

Mr. Crosse dwells strongly on the frequency with which a small calculus occasions retention of urine, a frequency so remarkable, that if a patient has retention without any very obvious cause, the surgeon should carefully endeavour to ascertain if a stone be not present in the bladder. In numerous instances of large vesical calculi requiring the operation of lithotomy, Mr. C. has learnt, by the previous history, that the patient had suffered sudden retention of urine at the time when the stone must have been small, and no doubt produced it, yet was not detected by the surgeon. Mr. Crosse relates a case, in which a patient was sent to him from a distance to undergo lithotomy. On sounding, Mr. C. ascertained, by attention to the circumstances so fully enumerated above, that the stone was of small dimensions. He introduced the urethro-vesical forceps constructed by Mr. Weiss, and readily removed a heart-shaped calculus, measuring one inch and three-quarters in its small circumference. The urethra was, of course, a capacious one.

But the surgeon must be satisfied that the stone is a small one, before he attempts its removal with the forceps. If too large to pass through the urethra, the attempt at extraction may be injurious, if not dangerous. In a case where persevering trials were made, the forceps continually slipping off the stone, on account of its being too large to be properly grasped by the instrument, or admitted to enter the urethra, such a severe degree of inflammation of the bladder ensued, as required most active antiphlogistic treatment to save the patient's life. To avoid such mistakes, Mr. Crosse points out the symptoms which determine the existence of a calculus too large to be removed by the urethra-forceps. If, then, the symptoms have steadily persisted, in a severe degree, for six or eight months—if the concussion of walking or riding produce pain in the glans penis or occasionally render the urine bloody—if there be a burning heat at the end of the penis, continuing some time after each evacuation of the bladder—if, when the bladder is empty, pressure above the pubes give a shooting pain in the glans penis; or when urine occupies the bladder, if the patient, on turning in bed from one side to the other, feel something move in its cavity; or if change of posture from lying to standing produce a sharp pain in the glans penis and neck of the bladder; if, in short, some or many of these symptoms be observed, but, more especially, if sounding comes to their support, by the dull noise occasioned, the firm resistance, and the extent of the surface touched; if these indications of a calculus of some size are elicited, the urethro-vesical forceps should on no account be introduced.

2. We may now suppose that the surgeon has been enabled to detect a small stone. His object is to remove it. Let us follow Mr. Crosse through his practical suggestions.

The situation of the stone, he says, suspected to be small enough for the use of such an instrument, should be first ascertained by a common sound, having the same length and curvature as the urethro-vesical forceps intended to be used; sometimes a bougie may be used to dilate the urethra or take off its irritability; and where there is any degree of stricture, it should be removed by proper treatment, as a preparatory step to the extraction of the calculus. He points out a disadvantage attending the common urethra-forceps—its allowing the urine to escape through the tube. The instrument should be so closely formed as to prevent the urine from running off in this manner. He suggests another improvement on the instrument, and thinks it may perhaps be better to have one blade longer than, and overlapping the other, which protects the mucous membrane of the bladder from the instrument, and equally prevents a small stone from slipping away, or from being fixed between the very extremity of the open blades.

" When the urethro-vesical forceps are introduced, they should not be made to project further into the cavity of the bladder than is necessary to feel the stone, before expanding the blades; if you project the instrument too far into the bladder, and the stone be small, it may lodge between the expanded blades, at too great a distance from their extremity, keeping the blades more expanded than is necessary, thereby creating difficulty, and inflicting pain, in the extraction, that might be avoided. If, on the other hand, the stone be held at the end of the blades, it will inevitably slip away when you attempt to bring it into the urethra. Feeling the stone at the extremity of the closed blades, you push these on a little, after expanding them, and endeavour to catch the calculus, being the position most advantageous for extracting it through the urethra." 60.

One quotation more, and we conclude this portion of the subject.

" If the stone, fairly grasped by the forceps, can be brought into the urethra, it will in general pass readily through the prostate portion of this canal, which offers the least resistance of any; and when it arrives in the membranous part, you may feel it *per anum* with the left forefinger, and if of considerable size, you can press it forwards, supporting it and preventing it slipping from the grasp of the blades; if very large, you may not be able to bring it any further than the membranous part of the passage, in which case rather than make violent attempts to do so, you should extricate the forceps from it, and keeping the left fore-finger curved beyond it *in ano* press it forward in the perinæum, and cut upon it by the gripe or Celsian method, or by a lateral incision as in lithotomy. If the calculus can be brought into the spongy part of the urethra, it has escaped the resistance of the most powerful muscles and also of the triangular ligament, and can be carried on to the orifice; I have, however, been obliged to cut calculi out, not only at the perinæum, but just anterior to the scrotum, finding it impracticable to bring them further forward, on account of their size, or having allowed the forceps to slip off and being unable to re-apply them. Under some circumstances, where you cannot get the calculus further onward, you may be glad to push it again back into the bladder, reserving it for an early future trial; but where practicable, it is better to cut the calculus out at once. Sometimes a stone, which has been readily made to traverse the rest of the urethra, cannot be made to pass the narrower and firmer orifice; when difficulty is experienced in this situation, rather than persist in using great force, it will be proper either to crush the stone, or to make an incision for its exit just behind the external orifice, whereby the patient will be spared much pain." 61.

Mr. Crosse concludes by relating the particulars of an interesting case. The patient had enlarged prostate, and his symptoms induced Mr. Crosse to believe that there were several small stones. In the course of two or three weeks, Mr. C. removed seven calculi by means of the urethra-forceps. After this, he found another stone, and was able to drag it into the membranous part of the urethra, but no farther. Here he was compelled to cut upon it. He pressed it forward with his left fore-finger *in ano*, and made a semilunar incision on the perinæal side of the anus, cutting down to the stone, which was placed an inch and a half from the surface; with a small scoop, he extracted the stone through the wound. It was an inch in length, and $\frac{13}{16}$ of an inch in breadth. Feeling another stone still in the bladder, he brought it to the wound by the urethro-vesical forceps, removed it, and found it a little smaller than the last. "I have met (he adds at the conclusion of the case) with many instances in which calculi have been extracted through the urethra with this useful instrument, but no other in which so large a stone as just described, has been brought into the urethra and cut out thence by the perinæum, which is very preferable to cystotomy, because a less dangerous operation, and one that every surgeon should be prepared to try on a fitting occasion, where by careful investigation he is led to believe that the stone is of such a size as to render the attempt advisable."

### Of Lithotrity.

The operation of lithotrity, or breaking up the stone in the bladder, occupies the ninth, a short chapter of the work. Yet Mr. Crosse has evidently no extensive experience of the operation, and an admission in a note presents the sum of all he has to offer. He is so fully impressed with the efficacy and advantages of the operation, that in any adult male patient with a stone small, yet too large to admit of being extracted by the urethra, he would not feel justified in recommending the more dangerous operation of cystotomy for its removal, unless he failed in previous attempts to crush it in the bladder. This so perfectly accords with the experience and the practice of most judicious surgeons, that we need not dilate upon the subject. Yet Mr. Crosse details a case which may usefully be glanced at.

*Case.* Mr. Crosse successfully lithotomized a man aged sixty-nine years. The patient remained well for ten years, when another calculus was found in the bladder. The *lithotriteur* of Civiale was used, and on a second trial a stone was grasped and reduced to fragments. For a few days, the symptoms of stone were absent, but they returned, and a stone could again be distinguished by the sound. "I repeated this examination (says our author) at the expiration of five weeks more, and observed 'that, on drawing the sound towards the pubes, I felt a calculus strike its concave aspect, causing a sharp sound, audible two or three yards off the patient, whilst, on jerking the sound towards the sacrum, I felt a calculus strike its convexity, giving a dull sound;' hence I entertained no doubt of there being two calculi of different sizes in the bladder." Before this examination, almost constant dribbling of the urine had come on, which persisted; rigors occurred, followed by parched tongue and feeble intermitting pulse; the abdomen became tumefied; and eighty days after the performance of lithotrity he died. One kidney was found to be healthy in appearance. The other presented the ordinary effects of long-continued suffering from vesical calculi—spurious hydatids in the cortical parts, dilatation of the pelvic cavity and infundibula; and absorption of much of the parenchymatous substance. The coats of the bladder were not much thickened, but on its cavity being exposed, a tumor, the size of a cherry, with a neck or narrowing at the basis, rose prominently from the left prostatic lobe into the bladder, and a similar tumor, much less prominent, was observed upon the corresponding part of the right lobe; these tumors were so placed as to be out of the way of the *lithotriteur*. Two calculi lay loose in the bladder, one weighing six drachms, the other one drachm and fifteen grains. In reference to these, Mr. Crosse observes—" they answered to the opinion I had formed at the last sounding, when the smaller stone, I have no doubt, lay on the pubic side of the instrument, giving the louder brisker sound, and the larger, giving a dull sound, rested towards the rectum."

### ON LITHO-CYSTOTOMY, OR CUTTING INTO THE URINARY BLADDER FOR THE REMOVAL OF A CALCULUS.

Mr. Crosse's own experience, and the practice of Mr. Martineau and of the Norwich Hospital which he has witnessed, must contribute to make surgeons attach some importance to his directions for the performance of this formidable operation. Yet he apologises, with more modesty than justice, for offering his sentiments and detailing his proceedings. He restricts his observations to the lateral operation, and points out the method of employing the curved and the straight staff. He feelingly insists on the necessity for caution in determining, or trying to determine, the size and characters of the stone, and in weighing the condition of the patient's health. On these points, however, we need not again touch, and we therefore proceed, without farther preface, to operate upon the patient. The following are Mr. Crosse's directions respecting the size and situation of the staff. The matter is of consequence, and the quotation rather long.

" The curved staff ought to be large enough to fill the urethra, the largest that can be admitted without great difficulty; it should be long enough to project an inch or two into the bladder, and with a deep semicircular groove upon the whole of its convex, and an adjoining portion of its straight part. The handle should be rough, and as it requires to be suited to the operator rather than the patient, it should be large, so as to receive the thumb and two fingers. The operator, feeling the staff in contact with the stone, inclines its handle a little to the right, that the curve may present *in perinæo* on the left side of the raphé; and satisfied with its position, he commits it to his assistant or staff-holder.

The staff-holder is the operator's main assistant, who should previously understand his views, and sympathize with him in every step of the operation. I have felt myself so dependant on such an assistant, that, preferring my fate to be in my own hands, I have sometimes wished to imitate Pouteau, by holding the staff for myself; but I have never undertaken to do so. This instrument must be held not forcibly, but lightly and as if suspended in air, since pressing it towards the sacrum to steady it, or pulling it towards the pubes, will equally tend to embarrass the operator and create mischief; it should be kept in the same relative position, in regard to the patient, as that in which it was received from the hands of the operator. Besides the danger of pressing the staff towards either the rectum or pubes, its holder, in endeavouring to make its convexity prominent in the perinæum, may cause its extremity to desert the bladder, so that it reach no deeper than the prostatic part of the urethra. By the unsteadiness of the patient, the staff may be moved irregularly from side to side, or backwards and forwards in the urethra; to prevent all this, the patient's pelvis must be kept steady, and if it move, there must be a corresponding movement of the staff, that it may retain the same relative position in regard to the patient's body." 72.

The operation itself, consisting, as it essentially does, of three incisions, is described by Mr. Crosse. He particularly insists on a free external one, the advantages of which must be obvious, while it is not attended by any inconvenience whatsoever. The second or middle incision, to be made by the common scalpel, should be of much less extent than the outer incision, answering to its middle third and taking the same direction; it is intended to divide the superficial perinæal fascia, the posterior part of the accelerator urinæ, and the left transversalis muscle, baring the staff in the membranous part of the urethra, just behind the bulb. The left fore-finger is, therefore, employed to feel for the staff, and to press the rectum out of the way, in order that it may not be wounded. The third stage of the operation, that of cutting into the bladder with the scalpel, is attended with most difficulty. The operator has to cut through the levator ani, lay open the remaining portion of the membranous urethra, and make an oblique section, outwards and downwards, of the left lateral lobe of the prostate gland. This is done by passing the knife on in the groove of the staff, supported by the forefinger of the left hand, and by enlarging the section of these parts in the same direction, downwards and outwards, as the knife is withdrawn. On the curved staff, Mr. Crosse has found this portion of the operation very difficult, in consequence of the instrument being held obliquely to present the groove favourably, and of the curve causing the instrument to recede in two directions from the operator. So much, then, for litho-cystotomy with the curved staff.

"The straight staff is calculated to obviate many of these difficulties, and possesses strong recommendations for a preference. It will be found an advantage to have a second handle, at a right angle with the usual one; the handle on a line with the body of the instrument serving for the operator, and the other for the staff-holder, to whom I venture to affirm it will be found very convenient, enabling him readily to keep the staff in contact with the stone, and maintain it in a fixed position in relation to the patient. The staff thus constructed and held, will follow every movement of the patient, without any tendency to displacement, so long as it is not suffered to go deeper into, nor to recede out of, the bladder.

In operating with the knife and straight staff, you execute the first and second stages of the incision as already described. The second handle is turned towards the right side, in order that the groove of the staff may be presented in the opposite direction; in doing this you find in the straight staff the great advantage of the instrument moving round its axis, and its position in the urethra consequently not altering from the median line, which I deem a very essential point. Although the urethra is naturally not straight, it readily yields so as to be made so, allowing a straight instrument to pass through it. The straight staff, in passing through the membranous part of the urethra, lifts it up from the rectum, pressing against the pubic or superior surface of the passage, thus affording great protection against wounding the rectum; the reverse happens with the curved staff, its convexity pressing towards the rectum, and rendering it not easy always to avoid wounding it. The greatest gain from the straight staff is in the facility given to the third stage of cutting into the bladder, by the instrument answering to the median line at the same time that its groove is presented in the most favourable position, and by your having to cut in a straight direction—so that getting down to the staff, you find this third stage converted into one plain continued incision, effected by carrying on the knife in the groove, as you would carry it along a common director, till satisfied that you have gone as deep as required, passing the prostate gland and just entering the bladder, you then enlarge the incision in withdrawing the scalpel." 74.

Mr. Crosse prefers the common scalpel to one with a more rounded end, as recommended by Mr. Key. He seems to hold the gorget in horror. In operating with the curved staff, Mr. Crosse advises, after the section of the bladder, the introduction of the blunt-beaked gorget as a conductor, and also as a dilator of the neck of the bladder. The finger, passed in upon the gorget, serves at the same time to distinguish the stone, and to dilate in some degree the neck of the bladder. But in operating with the straight staff, that is withdrawn as soon as the bladder is cut into, and before the finger is introduced by the wound.

" The forceps you select should vary in size according to the estimated bulk of the stone and age of the patient; it is requisite to select such forceps as do not meet at the end of the blades ; when the blades close, or nearly so, they are liable to catch hold of the coats of the bladder. The inner or concave surface of each blade should be rough, but not furnished with teeth, as I have seen some forceps ; for long projecting teeth are so many wedges for breaking a calculus, when the blades are pressed hard upon it. Whether you introduce the forceps upon the blunt gorget as a conductor, or without the blunt gorget, unless the deep incision has been very free, which is not always desirable, the narrowness of the deepest part of the wound affords resistance, and the forceps enter the bladder with a jerk, giving an impression by which the experienced hand knows well that the blades of the forceps are fairly in the vesical cavity : and when you find the forceps are so situated, you open their blades, and sweep the cavity of the bladder with them, by giving a quarter-turn of the instrument on its axis, and on closing the blades after so proceeding, you will, in a majority of cases, having a loose stone of moderate size to deal with, find it within their grasp, particularly if the stone have been felt with the forefinger as directed, enabling you to judge of its size and situation, and giving you a knowledge where it is to be found. Should the stone not be thus felt and laid hold of, you next use the closed forceps as a sound, feeling for the stone in different directions, before you again open their blades; and thus you proceed with repeated trials, until fortunate enough to grasp the stone.

When the curved forceps are required to be used, that you may be able to seize a stone placed at the *bas-fond* of the bladder, it will be found convenient to introduce them with the flat sides laterally directed, and the concavity towards the symphysis pubis, holding them at first nearly perpendicular, and bringing down the handles into a horizontal direction, as the blades pass under the arch of the os pubis; after the blades have thus reached the cavity of the bladder you give the instrument a half-turn, directing its extremity towards the rectum, where generally it is requisite to seek for the stone; it is in aged patients, and with the prostate enlarged, that the curved forceps are required, to enable the operator to get to a part of the vesical cavity, which he is generally unable to explore with the straight forceps." 76.

Mr. Crosse cautions the operator against employing force in the extraction of the stone. Force is said to be the instrument of the weak politician ; it is certainly that of the bad lithotomist. If great resistance opposes the exit of the calculus from the bladder, Mr. Crosse observes, that the assistant receives the forceps, and, standing over the patient as in holding the staff, pulls them forwards, still grasping the stone, as if to extract it, whilst the operator, with a curved probe-ended bistoury, cuts the resisting parts close to the stone, in the direction of the original wound, obliquely downwards and outwards, until relief is given. Sometimes he has known the resisting band felt upon the stone, on the side next the symphysis pubis; and the cut has, therefore, been made with the bistoury in that direction, or upwards. Mr. Crosse thinks it much better to enlarge the wound by a second cutting, at this part of the operation, when the size of the stone and the resistance met with prove the necessity for it, than to cut too freely at first, making a large deep wound (which is always dangerous), and securing a quick operation at the expense, perhaps, of security to the patient.

Even after the calculus has been brought into the wound, its removal is obstructed by too sparing a division of the levator ani muscle, or of the pubic ligament; or the forceps may slip off it. In either case, says Mr. C. after using the bistoury to enlarge the wound where resistance is found, you may pass the left forefinger into the rectum and curve it beyond the stone, supporting and pressing this forward, whilst with the rest of the same hand, you still assist the right in extracting the stone with the forceps.

Mr. Crosse observes in conclusion, and the observation should be carefully treasured up, that the quickest operators are not always most successful. Gentleness and precision are the principal requisites. Le Cat cut about half a dozen patients in twice as many minutes, and, it is said, lost nearly all of them. The final sentence is extremely just.

"After all, it is far easier to lay down rules for litho-cystotomy, than to execute them. The failures in this operation happen too often from the surgeon not acting as he intended, and not happening to cut precisely the parts which, in a plan or lecture, he would advise to be cut; this is particularly the case in operating with the scalpel, and I have repeatedly had opportunities of observing that the knife has not been carried deep enough, the anterior part of the prostate gland only being divided, sometimes not even that—and the rest of that body and the neck of the bladder being dilated or torn for the entry of the forceps, and still further injured in the forcible removal of the stone." 78.

### On the Treatment required after Litho-Cystotomy.

This is rather, perhaps, a chapter on the mode in which death occurs after lithotomy, or, as Mr. Crosse will have it, after litho-cystotomy, than absolutely a dissertation on the treatment generally required after the operation.

The ordinary management of an ordinary case need not surely occupy us. Mr. Crosse does not approve of leaving a canula in the wound and bladder, as a general rule. But if, in a few hours after the operation, the urine does not flow freely through the wound, and if the patient experiences uneasiness in the region of the bladder, which is felt distended above the os pubis, the finger ought to be introduced through the wound as a guide for a gum-elastic or silver female catheter, which may, indeed must, remain for some time. Mr. Crosse touches, and cautiously touches, on the employment of pressure to accelerate the healing of the perinæal wound, as soon as the urine flows freely through the penis. Yet this question, he thinks, is so made up of particular exceptions, that he rather refers to individual cases, than ventures to offer any decided general directions.

He successively reviews, as causes of death, or sources of danger after lithotomy, inflammation of the bladder—infiltration of urine into the cellular tissue, and diffuse inflammation of it—peritonitis, conjoined with the latter, and separate—and, finally, nervous exhaustion. He concludes the chapter by a few observations on tardy cicatrization or a fistulous condition of the perinæal wound—on wound of the rectum during the operation—and on fistulous opening into the gut.

1. Inflammation of the bladder, he remarks, seldom arises after litho-cystotomy, that organ being satisfied to have gotten rid of its offending inmate; the surgeon should however take care that it is not present when he undertakes the operation; and when it is detected, after the operation, by the acute pain in the viscus and tenderness on pressure upon it, active local and general antiphlogistic treatment should be adopted. In a case of this kind, which proved fatal, unattended by peritonæal inflammation, vomiting came on in addition to tension and great tenderness above the pubes, and the bladder on dissection was found particularly vascular and red in its inner membrane.

2. Diffuse inflammation of the cellular tissue of the pelvis, and abdomen, that is of its posterior region, is admitted by Mr. Crosse as the most frequent cause of death after the operation. Yet we think that he should have done Sir Benjamin Brodie the justice to allude to the fact, the unquestionable fact, that it is he who has earnestly, we had almost said exclusively, pointed out this important circumstance. Prior to the publication of that admirable surgeon's observations on the diseases of the urinary organs, the common occurrence of peritonitis after lithotomy, and the consequent necessity for active antiphlogistic treatment, were generally insisted on in books and lectures. The accurate observations of Sir Benjamin Brodie first and chiefly tended to dispel this vital and serious error. We do not think we need dwell any further upon this affection, as our readers will find in former numbers of this Journal the detailed remarks of Sir Benjamin Brodie, and a series of cases which ocurred at St. George's Hospital, reported by ourselves for the purpose of illustrating the characters of the disease.

3. Diffuse inflammation of the cellular tissue seldom proves fatal, seldom, perhaps, attains any considerable height without inducing some inflammation of the peritoneum. Yet that inflammation is sometimes indistinguishable, generally slight, and, even when severe, a secondary consequence.

It is doubtful if peritonitis ever occurs entirely independent of cellular inflammation. However this may be, it constitutes in some instances (the weak minority) the predominating affection, and may for practical purposes be considered as a distinct result of the operation. Modern observations have proved that as such it is not frequent.

The treatment of the two diseases is essentially different. For the first, active depletion appears to be injurious; for the second it is indispensably required. The surgeon should recollect that diffuse inflammation very often induces peritonitis, and probably local depletion is generally serviceable, both in the way of prevention and of cure. Our experience is in some degree opposed to that of Mr. Crosse in one respect. He says children are rarely sufferers from diffuse cellular inflammation. We have seen them not unfrequently die of it. We grant indeed that more or less inflammation of the peritoneum was present in every, or almost every instance. But the cellular inflammation was the most extensive, primary, and most essential malady.

3. Nervous exhaustion, unconnected with loss of blood, or with any well marked morbid condition of the tissues, is another cause of death after lithotomy. A tympanitic state of abdomen, says our author, with a feeble and easily compressed pulse, supervenes in adults, particularly in the aged, a few days after the operation, unaccompanied by pain, or the signs of peritonitis. Opiates are indicated, with good nutriment, sometimes administered by lavement, where the stomach is disinclined to receive it, and even stimulants are required; but bleed and blister in such a case, or omit to supply nutriment and stimulus, and the patient will have no chance of living. Danger is always to be apprehended, when such symptoms of nervous debility arise; but he has known recovery from the treatment recommended, where the abdomen was tympanitically distended, the pulse intermitting, and a troublesome hiccough present for some days.

Hæmorrhage after lithotomy is a fifth cause of death. But to this Mr. Crosse dedicates a separate, the last, chapter.

Mr. Crosse takes no notice of another, and a fatal consequence of the operation, though indeed it may occur independently of its performance—we allude to formation of circumscribed abscess in the pelvis. This not unfrequently occurs, and in general proves fatal. For a history of the affection, and some cases in which it was presented, we refer to the report from St. George's Hospital, to which we have already alluded.

4. The perineal wound sometimes heals slowly. Now, whenever a wound of any sort heals slowly, or shews a disposition not to heal at all, the philosophic surgeon either suspects that something is wrong in the general health, or something exists in the part itself to keep up irritation and prevent a cure. Perhaps there are few affections in which a rational treatment is more serviceable than in cases of fistulous wounds. One will not heal because there is a foreign body irritating it—another because a diseased structure is connected with it—a third, because a muscle is pulling on it—a fourth, because some fluid, as the urine or saliva, is flowing through it —in short, the causes of absence of cicatrization are numerous, and the judgment of the surgeon is displayed in the investigation of the circumstances, and appreciation of the one that is present in the individual case. The following are the remarks of Mr. Crosse on the subject of perineal fistula. Before we quote them, and as the only further commentary we shall make upon them, we repeat that the surgeon will display his judgment by minutely observing, or endeavouring to observe, what it is in the case immediately before him which seems to interfere with the healing process. Having thus discovered what is wrong, he must endeavour to set that right.

"The perinæal opening is often slow to heal after the operation, and one cause of it, I have found to be the feeble and reduced state of the patient, who with a lapse of a little more time, generous diet, and fresh air, has recruited in bulk and spirits, and quickly recovered. If the wound be patulous and a granulating surface present, a gum-elastic catheter may be introduced by the urethra and retained there; the urine passing through it and no longer by the wound, allows the latter to close. But there is much room for consideration before such a practice is adopted, and it is always pregnant with the one objection of expanding the urethra at the part where the wound is situated, and so far interfering with its cicatrizing. I do not consider the urine passing through the wound as the cause of its not healing, and advise the surgeon to look for some other. If, on the urine passing, there be pain, and matter escape from the wound, there is an unhealed cavity at the neck of the bladder, and it would be in vain to close the outward opening by pressure; and if the gum-elastic catheter be introduced into the bladder, and left in the urethra, great care must be observed in so doing, lest it get into the urinous cavity instead of the vesical.

When the perinæal opening is become very narrow, and is truly fistulous, by being lined with a cuticular membrane, if there be no sign of a larger and deeper cavity at the neck of the bladder, caustic may be applied to the fistulous orifice, the *argenti nitras,* or nitric acid; and if still unsuccessful, you should employ the actual cautery. It is however in vain, and therefore improper, to have recourse to these measures, unless, by observing the discharges from the opening, and examining by the probe, you have satisfied yourself, that there is no larger cavity at the neck of the bladder." 84.

5. Mr. Crosse has once only wounded the rectum. He has seen it wounded frequently, and so have we. The wound produced by the knife is constantly situated just above the sphincter ani, communicating with the membranous part of the urethra, and unless very small, the fæces, when soft, pass through it; and the urine passes into the rectum, of whatever size the opening. The rectum is so contiguous to the urethra, that an opening may occur subsequent to the operation, from sloughing resulting from violence, or from ulceration in a bad constitution.

When the wound is small, and the urine finds a free passage by the perinæal opening, healing will sometimes occur spontaneously, the motions being kept solid and purging avoided. Mr. Crosse once knew the wound of the rectum heal spontaneously a year and three-quarters after the operation; in this case the perinæal wound had closed early, a recto-urethral fistula only remaining. To treat this, its disclosure by means of the speculum ani, the paring of its edges, the application of the suture, or the use of lunar caustic, nitric acid, or the actual cautery, are familiar, and if the fistula is small, are sometimes not unsuccessful remedies. In one case, Mr. Crosse divided the sphincter ani also.

"Where the perinæal opening also remains, forming a recto-perinæo-urethral fistula, dividing the verge of the anus, by an incision including the parts between the two openings, has been recommended; I once succeeded by this method; in another case the perinæal opening only was closed by it, the communication between the rectum and urethra remaining. In a third case, a very emaciated subject, the parts all healed ultimately, after this procedure, on the health improving by country air and generous diet, so as to restore the wonted bulk and powers of the system." 84.

Mr. Crosse concludes by observing of wound of the rectum, that it disposes to a relapse of stone in the bladder. "This is so true (he says), that few with a recto-urethral fistula after litho-cystotomy escape a relapse; and when the operation is repeated for the second stone, it has cured the fistula left by the first. I have seen more than one such case, and read of many; whence we derive the suggestion of making an incision, similar to that of litho-cystotomy, to cure the ancient fistula." 85.

### ON HÆMORRHAGE AFTER LITHOTOMY.

This is a long chapter on an important subject. Hæmorrhage after any operation is formidable, not so much, perhaps, from the mere amount of the loss of blood, as from the disposition to many bad consequences, partly produced by the bleeding itself, partly resulting from the means required for its arrest. We have seen, of course, many instances of hæmorrhage after lithotomy. Too many of the cases ultimately ended ill—some from diffuse cellular inflammation—some from circumscribed abscess of the pelvis.

As Mr. Crosse observes, the causes of bleeding after the operation are various and cannot always be avoided.

"A very narrow pelvis and contracted perinæum will bring danger of wounding arteries of considerable size, in making an incision of proper extent and in the most eligible direction; or arterial branches, such as are of small size usually, and such as cannot be avoided, may be so enlarged, in particular cases, as to furnish an alarming bleeding; an unusual distribution of the arterial trunks about the neck of the bladder is a third source of hæmorrhage for which the surgeon should be prepared. But the supervention of hæmorrhage, after litho-cystotomy, most often depends on the incisions into the bladder not being practised exactly where intended by the operator and directed by the anatomist, whence vessels are wounded which are always large and regular in their distribution, and which ought to have been avoided. I can say little of hæmorrhage from particular diathesis of the patient, having never known a case of this kind; still it may no doubt occur, as we find it happening after other less serious operations, in those persons, the quality of whose blood and defective living powers of whose vessels, prevent the closure and stopping of the smallest divided arterial branch." 86.

Hæmorrhage may either be venous or arterial. The former being usually the slightest, may be first disposed of. It is not very unfrequent, and may generally be arrested when the operation is completed by moderate pressure and the recumbent posture. But in aged calculous patients the veins about the bladder, prostate gland, and rectum, are often very varicose; and in a feeble system, an abundant loss of venous blood, with some arterial, may prove fatal. In a gentleman much troubled with hæmorrhoids, on whom Mr. Crosse operated in his seventy-seventh year, removing two large calculi weighing nearly three ounces and a half, there occurred profuse loss of venous blood, with little of arterial, whilst he was on the table; bleeding, principally venous, continued after he was placed in bed, till at length Mr. C. introduced a gum-elastic canula, and round it plugged the wound with lint. The patient was already faint, and lay with a scarcely perceptible pulse for three or four hours. Cordials were freely given, and recovery took place.

Arterial hæmorrhage next occupies the attention of our author. He divides it into three varieties—as it occurs in the first, in the second, or in the third stage of the operation.

1. Hæmorrhage in the first stage, that is, in the performance of the first incision, arises from vessels in the adipose substance. The pressure of an assistant is generally sufficient to arrest it. But in a very feeble, or a very old patient, the vessel may be advantageously or necessarily tied.

2. Hæmorrhage in the second stage is of more consequence, and requires more elaborate consideration.

"As the left transversalis muscle must be divided, when an arterial branch of considerable size runs in the direction of it, the knife will unavoidably cut it across; but usually, although blood spirts, no trouble is occasioned, and the surgeon may proceed to finish the operation: the necessity of securing such a vessel must be determined by the quantity of arterial bleeding going on after the patient is in bed. A little bleeding in patients of good stamina should not excite alarm; I have known often from half a pint to a pint of coagulated blood on the sheet when first drawn, after the patient has been some time placed in bed, and no harm ensue. A moderate bleeding in strong plethoric subjects is indeed a great security against subsequent inflammation. It is the quick escape of fluid arterial blood from the wound, its being renewed instantly when removed by the sponge, and this occurring a few hours after the operation, that should be regarded as indicating a degree of hæmorrhage requiring the surgeon's interference for arresting it." 88.

If the hæmorrhage be rapid and alarming, the wound should be exposed in a good light, spatulæ employed to keep it open, and small sponges fixed upon a stick used to clean and clear its interior. With these precautions a ligature may be applied upon the bleeding vessel. But if it is impossible to distinguish the vessel or secure it, it is necessary to resort to plugging the wound. The plan adopted by Mr. Crosse is apparently of French extraction—the introduction of dossils of lint round a canula. A gum-elastic canula, he says, may be introduced into the bladder through the wound, and the latter be plugged up with dossils of lint, placed around the canula, beginning as deep as where the bleeding arises, and accumulating the dossils till they are on a level with the perinæum. Yet he properly insists on avoiding this coarse operation if possible. Whenever it is employed, he acknowledges that apprehensions of the final result must be felt, and that some untoward symptoms too constantly intervene. The bleeding may be arrested; that is not the danger. But the parts are irritated, the flow of urine is prevented, the natural progress of the actions of reparation is disturbed, and, as Mr. Crosse observes, inflammation of the cellular texture and peritonitis are liable to supervene. It is in cases where hæmorrhage has occurred, and the wound has in consequence been much disturbed, that we have mostly seen circumscribed abscess of the pelvis. Mr. Crosse relates a case illustrative of the indirect seriousness of hæmorrhage, and of the means required to control it.

*Case.* "In an aged gentleman, from whom I removed four calculi, by cystotomy after the lateral method, there was a brisk stream of arterial blood furnished from the wound, two hours after he had been placed in bed; and as I could not, on exposing the wound and turning out the coagula, detect the bleeding orifice for the application of a ligature, I stopped the hæmorrhage by pressing, for a quarter of an hour, the left internal pudic artery against the ramus of the ischium. I then introduced a gum-elastic tube by the wound into the bladder, (drawing off four or five ounces of dark urine,) and inserted dossils of lint, supporting the perinæum by a T bandage. The bleeding did not recur, and the patient went on seemingly well for a day or two, the urine flowing freely through the canula; but three days afterwards, the body became tympanitically distended, without pain or tenderness on pressure, and the pulse very feeble. Hot fomentations were applied, good animal broths and diluted wine also given, notwithstanding which the patient died eight days after the operation. Dissection proved the absence of peritonæal or other inflammation, as well as of urinary infiltration and suppuration. The whole of the colon, more especially its caput, was immensely distended with air; the stomach and small intestines were only moderately distended; to the most prominent part of the over-distended caput coli, the omentum was attached by recently effused lymph, and on being separated, a distinct round hole, the size of a split pea, was exposed in the coats of the bowel, through which fetid air escaped; this opening had been produced by sloughing or ulceration, so that the contents of the bowel were only prevented from escaping into the peritonæal cavity, by the adhering omentum. The inner surface of the colon, near this part, exhibited several ulcerated spots." 89.

Hæmorrhage from the arteries of the bulb is occasionally witnessed. Two vessels of some size, one on either side, enter the corpus spongiosum at this part. The left branch is naturally wounded most frequently, and the bleeding is considerable. The vessel may be seen and should be tied. In one case, mentioned by our author, where a troublesome arterial bleeding occurred at the bulb, it was stayed by compressing the right internal pudic artery on the ramus of the ischium, which was found practicable through the wound; hence it seemed that the operator had wounded the artery of the bulb on that side, whilst the left branch had escaped.

Although the operator *should* cut into the membranous part of the urethra, he not very unfrequently *does* open the bulb. Mr. Crosse has often seen this done where the wound was made too high, the surgeon going directly down to the staff where he could readily feel it. The wounded corpus spongiosum is secured by pressure and dry lint, and does not furnish an alarming bleeding, if the arterial branch be not divided before entering the bulb.

Mr.Crosse notices, almost par parenthése, the peculiarities of hæmorrhage in children. He makes two short remarks and adds one longer case. The remarks are these;—that young children rarely bleed much, the contractile powers of the vessels being great; but that when they do bleed, the hæmorrhage is badly borne. A slight one long continued may induce fatal syncope. The case is pregnant with instruction.

*Case.* A healthy boy, four years old, was lithotomized by the lateral method, with the scalpel and curved staff, and a small calculus removed with sufficient expedition. An hour and a half after the operation, the patient was cheerful, comfortable, and with cheeks florid as in health; an ounce or two of coagulated blood were on the draw-sheet at the first remove. In five hours, the responsible attendant on the spot, an intelligent surgeon, communicated that there was some bleeding going on, with swelled perinæum and rather pallid cheeks; but he took no alarm. Mr. Crosse proceeded to the house, but before he arrived the surgeon on the spot had been summoned up stairs by the nurse and found the child just dead. Mr. Crosse describes and exhibits a drawing of the parts. The bulb was wounded, and the left arterial branch entering it was wounded also. The fatal bleeding had been going on slowly, Mr. Crosse believes, between six and seven hours. He justly hints that the surgeon's carelessness was blameable. Had alarm been taken, the wound might have been exposed and the open artery secured by ligature; even pressure might have proved adequate to arrest the bleeding; or had a little stimulus, as well as nourishment, been given at the critical time, when the exhaustion induced had proved just sufficient to stop the bleeding, the powers of the system might have rallied and life been restored.

Having thus briefly noticed some circumstances connected with hæmorrhage in children, Mr. Crosse returns to the accident in adults. He points out the advantage and success in many cases attendant on pressure of the internal pudic artery against the ramus of the ischium. Such pressure may be kept up for several hours.

The internal pudic artery itself may be wounded in the third stage of cutting into the bladder. The hæmorrhage is of course of a formidable character, and must be speedily arrested. Mr. Crosse is convinced that no other means than a direct ligature will answer. Where the artery is divided quite across, a ligature above and below the division will be required to command the bleeding. He deems the internal pudic artery to be always accessible to a ligature, by means of a small curved needle, describing the third of a circle an inch or rather more in diameter, where the external wound is ample. A note contains a reference to three cases in which the pudic artery was tied. It is brief, and we shall introduce it.

" Dorsey (*Elements of Surgery,* vol. ii. p. 190, 3d edition,) gives a case in which Dr. Physic wounded the pudic artery with the gorget, and tied it with a ligature and curved tenaculum ; he also recommends a small curved needle, held by a pair of forceps. Mr. Abernethy mentions having tied the wounded pudic artery in the same situation as we compress it to stop the hæmorrhage. (*London Med. and Phys. Journal,* vol. ix, p. 393). An interesting paper, on hæmorrhage after lithotomy, is inserted in vol. iii, p. 292 of the *Quarterly Journal of Foreign Medicine and Surgery.* In a patient, æt. 57, bleeding went on till the pulse became imperceptible; the wounded pudic artery then tied and a cure in thirty-three days. *Castéra Diss. sur les Accidens qui compliquent la Taille,* p. 48)." 92.

Mr. Crosse adds a case in which he tied the artery without success. All facts connected with hæmorrhage after lithotomy are interesting. We will not therefore pass this by.

*Case.* " In the case of a gentleman, sixty-two years of age, of very irritable habit, there was a great arterial hæmorrhage, so that I calculated that a pint and a half of arterial blood was lost, in the delay of extracting several calculi, before the ligature was applied. The situation of the bleeding vessel was near the left ramus of pubis and ischium, half an inch from the commencement of the *erector penis,* which muscle was clearly exposed ; the blood flowed from a tube nearly as big as a crow's quill. Having made a very large external incision, I had no difficulty in seeing the spot whence the blood issued. I inserted a small tenaculum into the spot, and tied a ligature upon the part so held, when the bleeding immediately ceased and did not return. On the evening of the following day there was vomiting and hiccough, and the abdomen was prominent and distended with air, more especially in the region of the stomach and arch of the colon; there was neither pain, however, nor tenderness on pressure. The hiccough continued, the vomiting ceased, and urine flowed well through the wound; but the tongue became parched, pulse rapid and feeble, and death ensued five days after the operation. There was no inflammation of either peritonæum or any other structure, nor infiltration of urine, and I could only account for the fatal result, by the loss of blood, and the shock of the operation upon the nervous system of a most sensitive and irritable patient. The bladder and its appendages are represented in plate xxv. shewing the left internal pudic artery opened, but not cut across, on the aspect nearest to the wound, which accounts for a single ligature, applied by the aid of a tenaculum, stopping the bleeding. A divided artery of this size would require a ligature upon each orifice." 92.

Sometimes the internal pudic artery must be tied, not for a wound of the vessel itself, but of some arterial branch at a distance. Of course, if the bleeding orifice can be secured, that must be done.

*Case.* A man, aged 56, was in such bad health, that Mr. Crosse was prevented for a few weeks from attempting the operation. When he performed it, the prostate gland and neck of the bladder were divided by the scalpel. One superficial artery bled, and, in consequence of the feeble condition of the patient, Mr. Crosse secured it with a ligature. A stone, weighing nearly half an ounce was extracted. Within an hour, and before he left him, there was arterial bleeding to the amount of ten ounces, proceeding from the deepest visible part of the wound when he opened it to the utmost. He plugged the wound, but the bleeding continued, and at the expiration of three hours, the patient was cold, pallid, and exhausted, with a small and most rapid pulse, above one hundred and thirty in a minute. The bleeding orifice was not accessible, and arterial blood still flowing briskly, he opened the wound freely, cleared the bladder of coagulum by passing in his finger and the scoop, and then succeeded in putting a ligature on the left internal pudic artery, where it lies on the inner surface of the ramus of ossa pubis and ischii, by means of a small and very curved needle; the bleeding was instantly stopped and did not return. In the evening, the pulse were one hundred and twenty; there was sickness but no vomiting, and the urine flowed scantily by the wound. Next day the abdomen was tympanitic, and the distension was such as to occasion dyspnœa; there was also a constant desire to pass a motion, without an evacuation actually occurring. Cathartic pills, warm fomentations, and a glyster were the means employed by Mr. Crosse. The symptoms became favourable. On the fifth day, the superficial ligature separated—on the ninth, that on the pudic came away—on the tenth day, all the urine passed by the penis, and the perinæal wound uninterruptedly healed.

3. When an arterial branch is wounded in the third stage of the operation, and is situated deeper than the levator ani, either just on the inner surface of this muscle, or even on the face of the prostate gland, the orifice is probably quite inaccessible to the view, and a direct ligature cannot be applied. It will be right, says our author, to compress the left internal pudic artery, and observe if the bleeding be restrained by your so doing; but commonly an artery, thus deeply situated, does not branch off from the internal pudic, beyond the spot where the pressure is made; it may have quite a different course and another origin; and if the bleeding be, therefore, not restrained by pressing the internal pudic against the ramus of ischium and pubis, it would be in vain to put a ligature upon this vessel, and the only remaining resource is pressure in the deep part of the wound.

"When the bleeding has this deep origin, plugging the wound with lint around a hollow canula may be attended with bad consequences. You may stop the bleeding through the wound, but, the plugs not being driven deep enough, and not applied directly upon the bleeding orifice, the hæmorrhage continues and the blood gets into the bladder, which is known to happen, first by the state of the patient, who suffers from the loss—secondly by pain and distension of the bladder, and by fluid arterial blood flowing through the penis or through the canula. The blood may coagulate in the bladder, and if accumulated in large quantity, although the hæmorrhage cease, an embarrassing state of things is present, the urine not being allowed to flow through the canula. A syringe connected with the outer orifice of the canula, acting powerfully, will draw the coagulated blood through it, and clear the bladder; but in doing this, there is risk of the bleeding being reproduced. To make pressure in the deep part of the wound, under the state of things now considered, a hollow canula armed with compressed sponge may be introduced into the bladder, when the sponge, expanding from moisture, will act upon the surfaces required to be compressed; but all pressure, in such a deep situation in the pelvis, brings danger, and makes a serious case, creating apprehension of collateral bad effects—infiltration of urine, diffuse inflammation and suppuration: the fortunate circumstance is, that the cases requiring it are rare, particularly those arising from an extraordinary distribution of arteries at the neck of the bladder. It not unfrequently happens that some degree of bleeding takes place into the bladder; the urine, tinged as it flows, shews this; sometimes the bleeding is considerable, and yet there may be no ground for alarm, nor any alarm entertained. I knew a gentleman evacuate from the bladder a coagulum of five or six ounces of blood, through the wound, six days after the operation, with great straining and pain, after the manner of a woman in labour, and afterwards all went on well." 94.

Mr. Crosse does not feel inclined to imagine that hæmorrhage from the divided prostate gland, or from the injured lining membrane of the bladder, can ever prove serious from its amount, or make it necessary to consider formally the means of treatment.

Old writers on lithotomy talk of secondary hæmorrhage, occurring a week or two after the operation. This must prove the violence done in its performance. If no hæmorrhage occurs in the first six or eight hours, modern surgeons entertain little dread of its subsequent appearance. In two or three instances only has Mr. Crosse known bleeding to a considerable extent take place between a week and twelve days from the operation; one occurred in a little boy four years old, and all did well without the surgeon's interference. To open, as he remarks, the partly healed and greatly diminished wound, with the neighbouring soft parts swollen, tender, and inflamed, is impracticable at such a period: and so little chance is there of seeing the orifice of a bleeding vessel, that the surgeon is limited to making pressure in the wound.

Mr. Crosse concludes by the following short passage, equally apologetic for his essay and his practice. Yet neither requires an apology, for the former displays his industry, information, and candour, and the latter is at once the evidence and the reward of his ability.

"In summing up," he says, "this essay, at the close of the last of its chapters, I need not, I trust, explain the motives which have led me to state, so honestly and freely, whatever of untoward occurrences have happened, either in my own actual practice, or in the proceedings of others under my observation. To boast of uniform success in any capital operation, is not the dignified course of a surgeon, any more than that the physician should quack of universal cures. Experience in lithotomy, like victory in battle, is seldom gained, without counting a certain number of slain." 95.

We are sure Mr. Crosse's list of killed and wounded is but small. Ninety-five pages of the volume are consumed in the matter of which we have given, not merely an epitome, but almost an impression. The reader of a journal must surely be more satisfied at finding himself the possessor of the substance of a valuable volume, than at reading some pert criticisms, imperfect commentaries, and inferior jokes. The one is useful knowledge—the other idle and ephemeral trash.

The succeeding forty pages are occupied with the description of twenty-nine coloured lithographic plates. Those plates are principally devoted to the delineation of preparations in the possession of Mr. Crosse. The execution is respectable—the representation, we may presume, is accurate—and the study of the plates will repay the time and attention of the surgeon.

Three Appendices conclude the volume. The first, consisting of thirty pages, is occupied with twenty-two cases; the second with ten tables, constructed with labour, and no doubt with accuracy. In order to render our view of the present volume complete, we shall insert the majority of the tables here, and in our clinical department we shall fully notice the more interesting cases.

The third appendix must have cost the author most extraordinary labour. It is a catalogue of the express treatises upon gravel, stone, and lithotomy, published in different ages and countries, and of essays or notices referring to those subjects in many periodical works. This catalogue is for reference, and forms an imposing monument of the industry of Mr. Crosse.

### Tables shewing the Results of Operations, chiefly performed at the Norfolk and Norwich Hospital.

These tables are constructed from the collection in the hospital. From the first opening of the institution, so Mr. Crosse tells us, to the present period, embracing above sixty years, all urinary calculi, removed from the human bladder by operation, whether on male or female patients, have been preserved and are now contained in a cabinet, arranged in numerical and chronological order, with an inscription to each, distinguishing the name of the operator, the name and age of the patient, the weight of the calculus removed, the day and year of the operation, and the date of the patient's discharge as cured, or of his death, where the result was unfavourable.

The first table contains a list of the calculi preserved in the cabinets of the hospital. The number appears to be seven hundred and four. The age of the patient—the result—the length of time intervening between it and the performance of the operation—and the weight of the stone are told. The number of females operated on is thirty-five. This table is too long for insertion here.

The other tables are analyses of the preceding, containing the most important averages, details, and deductions. These we shall insert.

### "TABLE II.

The seven hundred and four cases of litho-cystotomy, specified in table 1, are here arranged so as to shew the number of cured and of fatal cases, and the proportion of the one to the other, with a distinction of male and female.

| MALE. | | | FEMALE. | | | TOTAL. | | |
|---|---|---|---|---|---|---|---|---|
| OPERAT. | CURED. | DIED. | OPERAT. | CURED. | DIED. | OPERAT. | CURED. | DIED. |
| 669 | 578 | 91 | 35 | 33 | 2 | 704 | 611 | 93 |
| 1 in $7\frac{32}{91}$ | | | 1 in $17\frac{1}{2}$ | | | 1 in $7\frac{53}{93}$ | | |

### TABLE III.

Seven hundred and four cases of litho-cystotomy, according to the list in the collection of calculi at the Norfolk and Norwich Hospital, and as shewn in table 1, arranged in decennial periods as to age, with a distinction of the numbers cured or ending fatally.

| Years. | 1 to 10 | | 11 to 20 | | 21 to 30 | | 31 to 40 | | 41 to 50 | | 51 to 60 | | 61 to 70 | | 71 to 80 | | TOTAL. | |
|---|---|---|---|---|---|---|---|---|---|---|---|---|---|---|---|---|---|---|
| Result. | C | D | C | D | C | D | C | D | C | D | C | D | C | D | C | D | C | D |
| Numb. | 262 | 19 | 97 | 9 | 43 | 5 | 45 | 5 | 37 | 10 | 71 | 25 | 50 | 20 | 6 | 2 | 611 | 93 |
| Propor. | 1 in $14\frac{15}{19}$ | | 1 in $11\frac{7}{8}$ | | 1 in $9\frac{3}{5}$ | | 1 in 16 | | 1 in $4\frac{7}{10}$ | | 1 in $3\frac{21}{25}$ | | 1 in $3\frac{1}{2}$ | | 1 in 4 | | 1 in $7\frac{58}{93}$ | |

### TABLE IV.

Seven hundred and four calculi preserved in the cabinet of the Norfolk and Norwich Hospital, arranged according to their weight in ounces, with distinction of the result of the operations performed for their removal.

| 1 oz. &un- | | 1 to 2 oz | | 2 to 3 oz. | | 3 to 4 oz. | | 4 to 5 oz. | | 5 to 6 oz. | | 6 to 7 oz. | | 7 to 8 oz. | |
|---|---|---|---|---|---|---|---|---|---|---|---|---|---|---|---|
| C | D | C | D | C | D | C | D | C | D | C | D | C | D | C | D |
| 482 | 47 | 101 | 18 | 19 | 16 | 4 | 7 | 2 | 3 | 2 | 0 | 0 | 2 | 1* | 0 |

* This calculus, marked No. 37 in table 1, and weighing just eight ounces, I have reason to believe was contained in the scrotum, and the case, therefore, cannot be regarded as a successful example of regular cystotomy.

### TABLE V.

Five hundred and twenty-nine calculi, removed by cystotomy, weighing *one ounce or under*, taken from the first division of the preceding table, and arranged according to their weight in drachms, with the result as to the cure or death of the patient. Forty-seven out of the number being fatal cases, the proportion of deaths is *one* in about *eleven and a quarter*.

| 1 dr&un. | | 1 to 2 dr. | | 2 to 3 dr. | | 3 to 4 dr. | | 4 to 5 dr. | | 5 to 6 dr. | | 6 to 7 dr. | | 7 to 8 dr. | | TOTAL. | |
|---|---|---|---|---|---|---|---|---|---|---|---|---|---|---|---|---|---|
| C | D | C | D | C | D | C | D | C | D | C | D | C | D | C | D | C | D |
| 122 | 12 | 101 | 10 | 90 | 5 | 60 | 8 | 28 | 1 | 35 | 3 | 21 | 3 | 25 | 5 | 482 | 47 |

### TABLE VI.

A list of one hundred cases of death after litho-cystotomy in males, shewing the age in years of each patient, the weight of the calculi, and the interval after the operation at which the fatal event ensued. The first ninety-one patients of this list ar^ taken from table 1, the rest are supplied from my notes, being cases which I witnessed, or in which I operated in private practice. In eighty-four out of the hundred cases there was a solitary calculus; in seven cases there were *two* calculi, in six there were *three*, in two there were *four*, and in a single instance *five*.

| No. | Age. | WT. OF CAL. | INTERV. | No. | Age. | WT. OF CAL. | INTERV. | No. | Age. | WT. OF CAL. | INTERV. |
|---|---|---|---|---|---|---|---|---|---|---|---|
| 1 | 5 | 8 grains | 12 days | 35 | 62 | 1 ounce | 22 days | 69 | 50 | 7 drms. 20 rgs. | 2 days |
| 2 | 45 | 1 oz. 4 drms. | 7 days | 36 | 5 | 30 grains | 2 days | 70 | 16 | 6 drms. 30 grs. | 29 days |
| 3 | 61 | 2 ounces | 107 days | 37 | 49 | 6 drachms | 18 days | 71 | 66 | 1 oz. 5 drms. | 4 days |
| 4 | 59 | 2 oz. 4 drms. | 91 days | 38 | 40 | 2 ozs. 4 drms. | 5 days | 72 | 5 | 4 drms. 20 grs. | 10 days |
| 5 | 68 | 5 ounces | 15 days | 39 | 2 | 1 drachm | 17 days | 73 | 52 | 3 ounces | 18 days |
| 6 | 45 | 1 oz. 1 drm. | 21 days | 40 | 14 | 10 grains | 25 days | 74 | 66 | 4 drachms | 20 days |
| 7 | 8 | 1 oz. 2 drms. | 11 days | 41 | 51 | 3 drms. 30 grs. | 6 days | 75 | 62 | 3 ounces | 3 days |
| 8 | 26 | 2 oz. 4 drms. | 18 days | 42 | 61 | 1 oz. 5 drms. | 28 days | 76 | 70 | 2 oz. 4 drms. | 6 days |
| 9 | 3 | 1 drachm | 1 day | 43 | 59 | 2 oz. 3 drms. | 31 days | 77 | 40 | 3 drachms | 14 days |
| 10 | 29 | 3 drachms | 51 days | 44 | 57 | 6 drms. 30 grs. | 40 days | 78 | 64 | 1 drm. 2 scruples | 32 days |
| 11 | 3 | 20 grains | 23 days | 45 | 17 | 2 oz. 3 drms. | 23 days | 79 | 65 | 2 ounces | 5 days |
| 12 | 50 | 4 oz. 4 drms. | 2 days | 46 | 7 | 4 drachms | 89 days | 80 | 51 | 1 drm. 30 grains | 29 days |
| 13 | 42 | 2 oz. 4 drms. | 2 days | 47 | 25 | 2 oz. 4 drms. | 17 days | 81 | 63 | 1 oz. 6 drms. | 4 days |
| 14 | 54 | 2 oz. 4 drms. | 25 days | 48 | 46 | 2 drachms | 2 days | 82 | 47 | 1 drm. 20 grains | 20 days |
| 15 | 54 | 3 oz. 4 drms. | 41 days | 49 | 51 | 2 ounces | 1 day | 83 | 65 | 6 oz. 4 drms. | 3 days |
| 16 | 59 | 2 oz. 4 drms. | 49 days | 50 | 5 | 3 drms. 30 grs. | 15 days | 84 | 60 | 1 drm. 36 grs. | 6 days |
| 17 | 67 | 4 oz. 4 drms. | 4 days | 51 | 60 | 3 ounces | 66 days | 85 | 4 | 1 drachm | 21 days |
| 18 | 66 | 3 drachms | 13 days | 52 | 4 | 1 drm. 40 grs. | 4 days | 86 | 64 | 3 oz. 6½ drms. | 4 days |
| 19 | 12 | 1 oz. 4 drms. | 5 days | 53 | 71 | 1 ounce | 2 days | 87 | 4 | 12 grains | 7 hours |
| 20 | 60 | 3 ounces | 21 days | 54 | 4 | 10 grains | 2 days | 88 | 68 | 1 oz. 1 drachm | 12 days |
| 21 | 60 | 1 ounce | 2 days | 55 | 67 | 1 oz. 6 drms. | 23 days | 89 | 75 | 3½ oz. 2 scruples | 8 days |
| 22 | 57 | 4 ounces | 3 days | 56 | 5 | 3 drachms | 16 days | 90 | 3 | 1 drachm | 20 days |
| 23 | 40 | 2 ounces | 11 days | 57 | 50 | 3 oz. 4 drms. | 4 days | 91 | 51 | 6 drms. 24 grs. | 10 days |
| 24 | 19 | 1 ounce | 19 days | 58 | 22 | 6 drachms | 6 days | 92 | 43 | 13 ounces | 4 hours |
| 25 | 51 | 1 oz. 2 drms. | 12 days | 59 | 57 | 4 drachms | 2 days | 93 | 68 | 5 drms. 20 grs. | 4 days |
| 26 | 61 | 2 drachms | 10 days | 60 | 59 | 3 drms. 30 grs. | 7 days | 94 | 56 | 6 drms. 40 grs. | 13 days |
| 27 | 54 | 2 drachms | 6 days | 61 | 53 | 1 oz. 2 drms. | 7 days | 95 | 65 | 1 oz. 2½ drms. | 7 days |
| 28 | 70 | 3 ounces | 8 days | 62 | 8 | 16 grains | same day | 96 | 67 | 1 oz. 3½ drms. | 36 hours |
| 29 | 57 | 2 ounces | 6 days | 63 | 3 | 40 grains | same day | 97 | 62 | 1 oz. 1½ drm. | 5 days |
| 30 | 30 | 7 ounces | same day | 64 | 67 | 1 drm. 30 grains | 15 days | 98 | 69 | 2 oz. 50 grains | 37 days |
| 31 | 45 | 4 drachms | 17 days | 65 | 16 | 6 drachms | 5 days | 99 | 72 | 5 drachms | 8 days |
| 32 | 16 | 1 oz. 2 drms. | 13 days | 66 | 3 | 4 drachms | 1 day | 100 | 55 | 3 drms. 20 grs. | 7 days |
| 33 | 15 | 1 oz. 5 drms. | 13 days | 67 | 54 | 4 ounces | 2 days | | | | |
| 34 | 53 | 2 oz. 2 drms. | 6 days | 68 | 9 | 2 drms. 50 grs. | 1 day | | | | |

### TABLE VII.

The calculi in one hundred fatal cases of litho-cystotomy, as in the preceding table, arranged according to their weight in ounces.

| 1 oz.&un. | 1 to 2 oz. | 2 to 3 oz. | 3 to 4 oz. | 4 to 5 oz. | 5 to 6 oz. | 6 to 7 oz. | abv. 7 oz. |
|---|---|---|---|---|---|---|---|
| 50 | 22 | 17 | 5 | 3 | 0 | 2 | 1 |

### TABLE VIII.

Twelve cases of males who underwent litho-cystotomy a second time, shewing the age *in years* of each patient at the time of his being first operated on, the interval *in months* between the first and second operation, and the weight of the calculi removed. Two of the patients (No. 9 and 11) died from the second operation, the rest recovered; one patient (No. 3) had stone a third time, and was deemed unfit for the operation.

| No. | Age at 1st Operation. | Interval between 1st and 2d Operation. | Weight of Calculi. 1st Operat. | 2d Operat. | Observations. |
|---|---|---|---|---|---|
| 1 | 15 | 16 months | ʒij. | ʒiij. | Three calculi removed at the first operation, broken into many fragments. |
| 2 | 48 | 12 months | ʒiij. | ʒv. | One flat lithic acid calculus first removed, entire; the calculus removed by the second operation is of the same composition. |
| 3 | 63 | 32 months | ʒiv. | ʒiij. | One flat lithic-acid calculus at first operation, unbroken. |
| 4 | 26 | 12 months | — | ʒiv. | Weight of the first stone unknown; both rectal and perinæal fistulæ from the first operation, which were cured by the second. |
| 5 | 8 | 17 months | ʒij. ℈j. | ʒivss. | One entire calculus at first operation, which left a recto-urethral fistula. |
| 6 | 3½ | 24 months | ʒiiss. | ʒij. ℈ij. | The first calculus removed entire. |
| 7 | 2½ | 15 months | ℈iiss. | ʒj. | The first calculus alkaline, and broken into many fragments in the extraction. |
| 8 | 3 | 14 months | ℈j. | ʒiiss | Two small calculi of oxalate of lime removed by the first operation. |
| 9 | 65 | 130 months | ℥iss. | ℥ij. | The first stone entire; the second was an oval stone, composed principally of lithic-acid. |
| 10 | 18 | 8 months | ʒvj. | ʒivss. | The first stone was entire with an alkaline exterior. |
| 11 | 46 | 12 months | ʒxss. | ʒj. ℈j. | The rectum wounded in the first operation, and a recto-urethral fistula present when the second was performed. |
| 12 | 7 | 24 months | ʒiss. | ℈ij. | The first operation was perfect, and a firm entire globular calculus removed. |

Here we close our account of the present volume. That it evinces the patience, the labour, and the zeal of Mr. Crosse must be evident. Its principal merit is connected with the operation of lithotomy, and the pathological alterations of the urinary organs. The chemistry, and what may be denominated the medical surgery of those organs, is left comparatively unnoticed and unimproved. It is a work which all hospital surgeons will possess—indeed, which all surgeons who wish to be well acquainted with their profession should. The copiousness of our notice is sufficient evidence of our practical estimate of its deserts, and we wish the author all the fame and profit which he merits, no inconsiderable store.

